# MS‐based multi‐dimensional metabolomics reveals protective effect of Polygalae Radix against metabolic disturbances in Alzheimer's disease mice

**DOI:** 10.1002/ctm2.70292

**Published:** 2025-04-15

**Authors:** Yanwen Chen, Lisha Zhao, Shuo Cai, Yuchen Zou, Weiwei Tang, Bin Li

**Affiliations:** ^1^ State Key Laboratory of Natural Medicines and School of Traditional Chinese Pharmacy China Pharmaceutical University Nanjing Jiangsu Province China

1

Dear Editor

The system‐wide metabolic dysregulation is a central hallmark of Alzheimer's disease (AD).^1^, ^2^, ^3^ Growing evidence indicated that Polygalae Radix (PR) has ameliorative effects on memory deficits of AD.^4^, ^5^ However, the anti‐AD effect of PR extract (PRE) via regulating metabolic disturbance is seldom investigated from the perspective of a system‐wide level. In this study, matrix‐assisted laser desorption/ionization mass spectrometry imaging (MALDI–MSI)‐based spatial metabolomics and liquid chromatography‐mass spectrometry (LC–MS)‐based metabolomics were applied to explore the anti‐AD effect of PRE via improving the system‐wide metabolic disorders in APPswe/PSEN1dE9 (APP/PS1) double transgenic AD model mice.

The chemical composition of PRE was first characterised by ultra‐performance liquid chromatography‐tandem mass spectrometry (UPLC–MS/MS) (Figure S1 and Table S1). The results of behavioural and pharmacological experiments indicated that PRE treatment remarkably ameliorated the impairment of learning and memory in AD model mice, and significantly reduced the levels of amyloid‐β (Aβ) plaques (Figure S2) and Aβ_1–42_ monomers, and increased the levels of acetylcholine and brain‐derived neurotrophic factor in the cerebral cortex (Ccx) and hippocampus (Hp) of AD mice (Figure S3) (see the Supporting Information for details). Subsequently, MALDI–MSI was utilised to investigate the ameliorative effect of PRE against regional metabolic disturbances in AD mouse brains. Principal component analysis (PCA) and partial least squares discrimination analysis (PLS‐DA) showed that the metabolic profiles in Ccx, Hp, corpus callosum (Cc), thalamus (Th) and hypothalamus (Hth) of PRE‐treated mice were remarkably distinguished from AD, indicating that notable spatial metabolic disturbances could be changed by PRE (Figures 1A and S4). Tissue‐specific differential metabolites between PRE‐treated and AD model mice were identified in five brain regions. In Ccx, 59 differential metabolites were observed, and the levels of 27 out of 59 returned to control (CON) after PRE treatments (Figure 1B‐1C and Table S2). The spatial distribution and level changes of representative metabolites in CCx before and after PRE treatment are shown in Figure 1C. Similarly, a total of 75, 59, 56 and 53 differential metabolites between the PRE‐treated and AD model groups were identified in Hp, Th, Hth and Cc regions, respectively (Figure S5 and Tables S3–S6). Among them, the levels of 39 differential metabolites in Hp, 23 in Th, 23 in Hth and 19 in Cc had been reversed after PRE treatments (Figures S6 and S7). Therefore, MALDI–MSI results revealed that PRE treatment could regulate the drastic brain metabolic disturbances with a critical spatial metabolic heterogeneity (Figure 1D). According to KEGG reference pathways, PRE treatment regulated the imbalance of multiple metabolism pathways at different levels in five brain regions (Figures 1E, S8 and S9). A partial metabolic pathway network of selected significantly altered metabolites was mapped (Figure 2), demonstrating that PRE treatment corrected spatial metabolic disturbances in the AD model mouse brain in a region‐specific manner.

**FIGURE 1:**
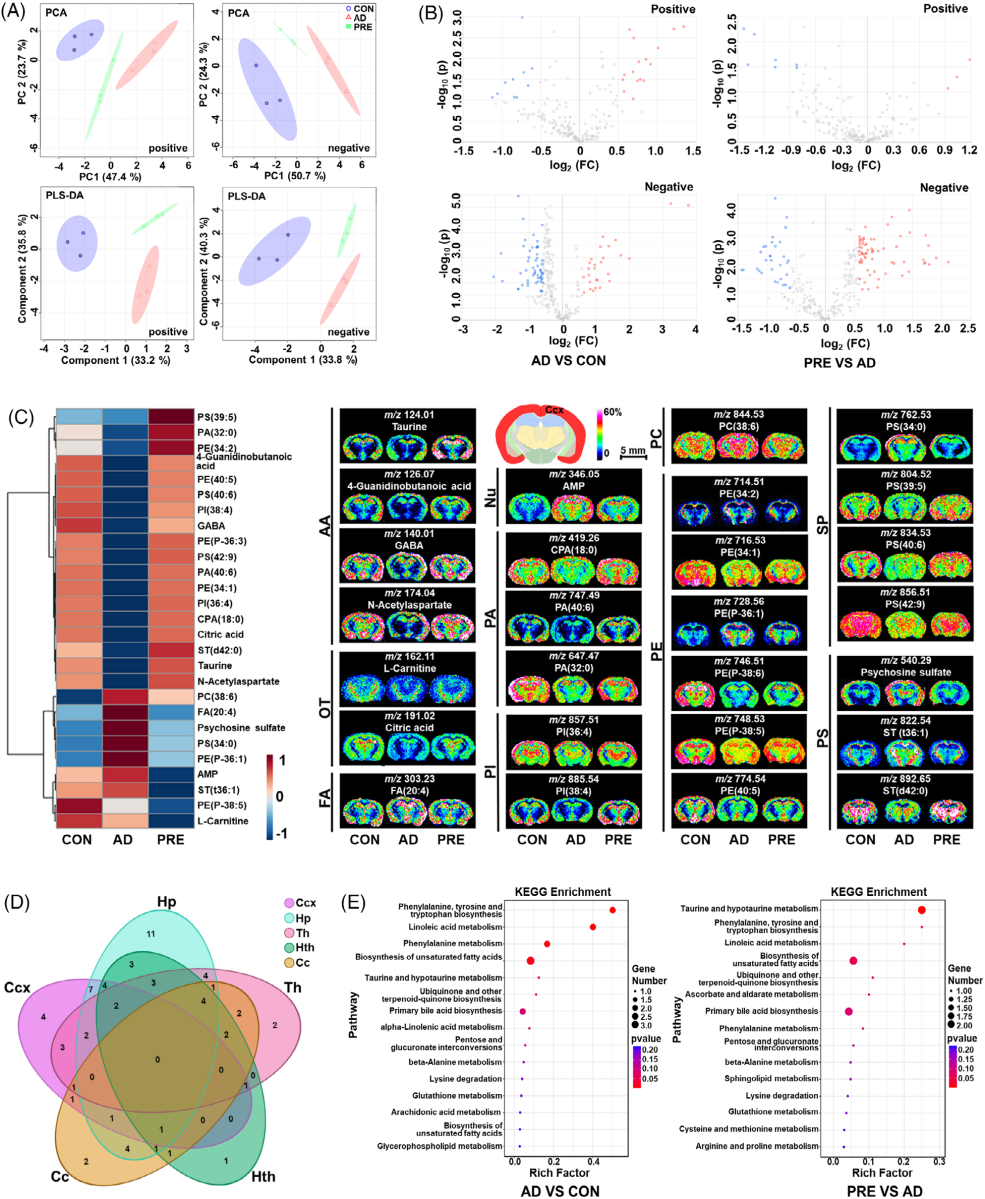
Regulatory effects of PRE on the brain region‐specific metabolic disturbances in AD model mice. (A) PCA score plot and PLS‐DA score plot of Ccx region derived from MALDI‐MS data obtained in negative and positive ion modes, respectively. (B) The volcano plot displayed differential metabolites with fold‐change > 1.5 in the Ccx region between the AD and CON groups as well as PRE‐treated and AD groups, respectively. (C) Heat map and ion images of differential metabolites reversed by PRE in the Ccx of AD model mice. (D) Venn diagram of reversed metabolites after PRE treatment in Ccx, Hp, Th, Hth and Cc. (E) The enriched metabolic pathways for the differential metabolites in Ccx of AD and PRE‐treated mice. All MALDI–MSI data were recorded with a step size of 200 µm. Ccx, cerebral cortex; Hp, hippocampus; Th, thalamus; Hth, hypothalamus; Cc, corpus callosum; AA, amino acids; FA, fatty acids; OT, others; Nu, nucleotides; PA, phosphatidic acid; PI, phosphatidylinositol; PC, phosphatidylcholine; PE, phosphatidylethanolamine; SP, sphingolipids; PS, phosphatidylserine. The value of colour scale bars represents the relative intensities (*n* = 3 per group).

**FIGURE 2:**
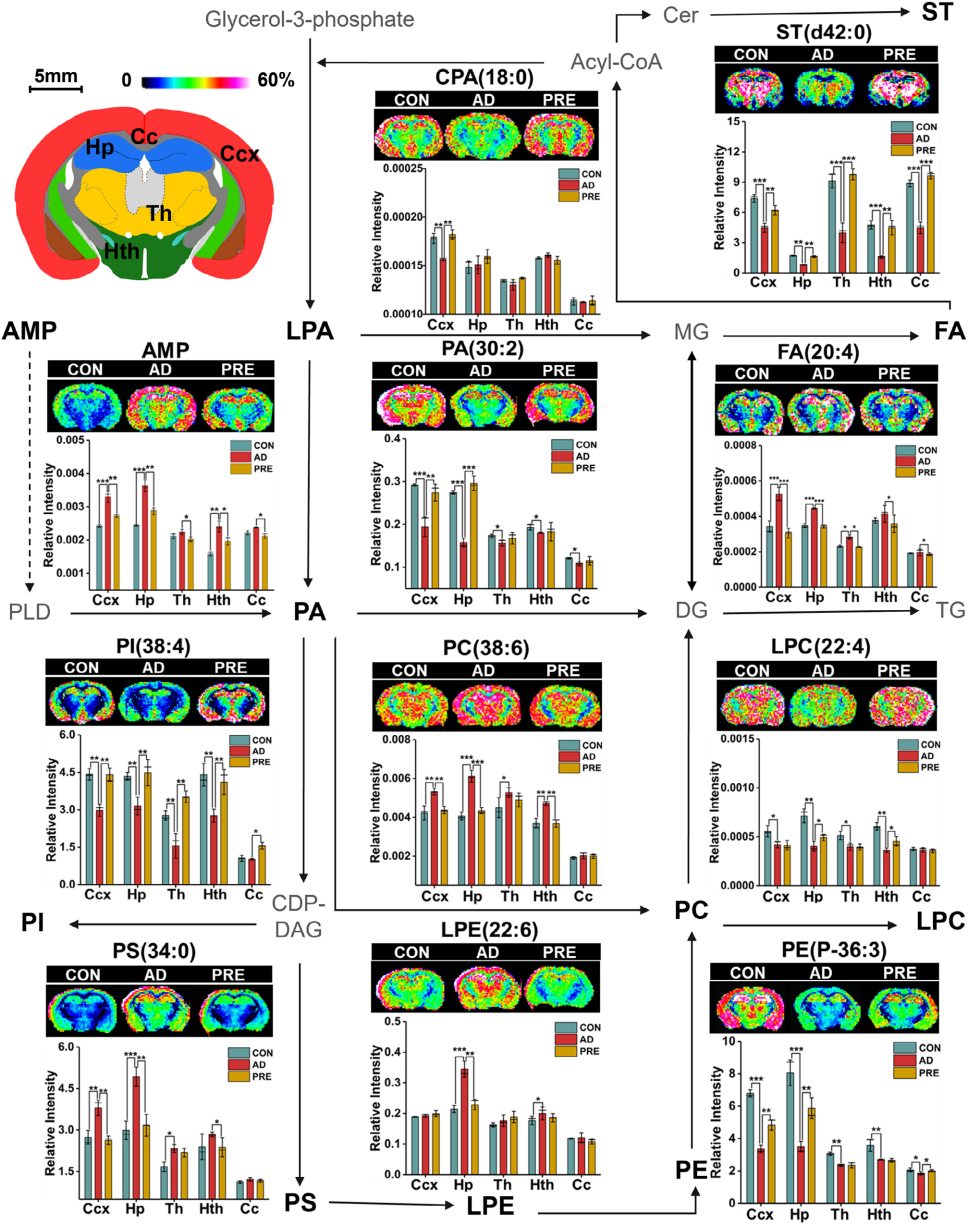
The partial metabolic network map and the spatial distributions of the differential metabolites reversed by PRE obtained from the coronal brain sections of three groups using MALDI–MSI. All MALDI–MSI data were recorded with a step size of 200 µm. All data are presented as the mean ± SEM (*n* = 3 mice per group). *p < .05, **p < .01, ***p < .001. Ccx, cerebral cortex; Hp, hippocampus; Th, thalamus; Hth, hypothalamus; Cc, corpus callosum. The value of colour scale bars represents the relative intensities.

Moreover, blood, urine and faeces were analysed using UPLC–MS/MS to investigate the biofluid metabolic changes. PCA and PLS‐DA results revealed three distinct groups of blood (Figure 3A). Similar results were observed in urine and faeces (Figure S10). OPLS‐DA results showed that 41 differential metabolites in blood were identified between PRE‐treated and AD groups, 43 in urine and 42 in faeces. PRE treatment reversed the abnormal levels of 28 differential metabolites in the blood, 33 in urine and 24 in faeces (Figures 3B and S11–S13 and Tables S7–S9). The similarities and differences of the reversed metabolites in blood, urine and faeces are shown in Figure 3C. KEGG pathway enrichment results showed that PRE treatment regulated the linoleic acid metabolism, alpha‐linolenic acid metabolism, ether lipid metabolism to ameliorate metabolic disturbance in the blood of AD mice (Figure 3D). In urine, the disturbed pathways, including valine, leucine and isoleucine biosynthesis, aminoacyl‐tRNA biosynthesis and phenylalanine metabolism, could be corrected by PRE treatments, and in faeces the disturbed pathways, including taurine and hypotaurine metabolism, phenylalanine, tyrosine and tryptophan biosynthesis, linoleic acid metabolism, could be corrected (Figure S14).

**FIGURE 3:**
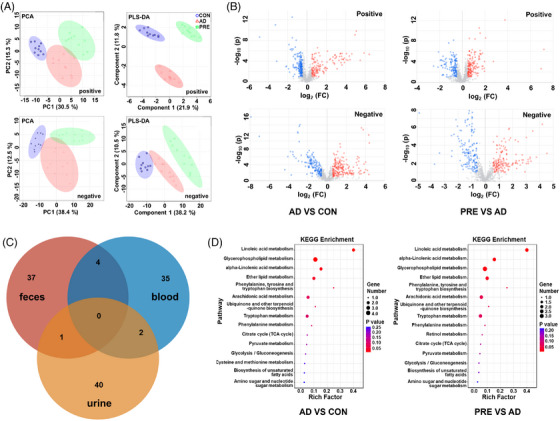
Regulatory effects of PRE on the metabolic disturbances in the blood of AD model mice. (A) PCA and PLS‐DA score plots of blood samples derived from UPLC Q‐TOF MS/MS data obtained in negative and positive ion modes, respectively. (B) Volcano plots displayed differential metabolites in blood with fold‐change > 1.5 between AD and CON groups as well as between PRE‐treated and AD groups, respectively. (C) Venn diagram of PRE reversed metabolites in blood, urine and faeces. (D) The enriched metabolic pathways for the differential metabolites in the blood of AD and PRE‐treated mice (*n* = 10 per group).

Finally, the Spearman‐rank correlation matrix of five brain regions was plotted to investigate region‐specific metabolic differences among three groups. In the CON group, high positive correlations were observed between Ccx and Hp (*r *> 0.8, *p *< .05) and moderate correlations between Cc and Th, Ccx and Hth, Hp and Hth (0.6 < *r *< 0.8, *p *< .05). However, the correlations among the Hp, Ccx, Th and Cc were totally altered in AD model mice. Notably, the high positive correlations between Ccx and Hp were recovered after PRE treatment and the moderate correlations between Hp and Cc, Hp and Th diminished (Figure 4A). Among five brain regions, Hp of AD model mice showed the most drastic change of regional metabolism in terms of the number of altered metabolites and metabolic pathways, followed by the Ccx (Figure 4B). The metabolite–metabolite correlation matrices among the three groups showed that AD model mice exhibited a notable reduction in both high positive and negative correlations among metabolites. Furthermore, there was a significant alteration in correlation patterns in PRE‐treated mice (Figure S15). For example, the level of taurine was positively correlated with that of N‐acetylaspartate in the Ccx and Hp of CON mice (*R* = 0.697 and 0.657, respectively), but the correlation was severely diminished in the AD model group (*R* = 0.334 and 0.380, respectively), whereas the correlation was significantly recovered in PRE‐treated group (*R* = 0.669 and 0.625, respectively) (Figures 4C and S16). Furthermore, metabolic networks were constructed for each brain region, with nodes representing metabolites and edges representing correlations (*R* > 0.90) (Figure 4D). The results were consistent with metabolite–metabolite correlation matrices in which the altered correlations in AD model mice were significantly changed by PRE treatment (Figures 4D and S15). To evaluate the system‐wide metabolic homeostasis, the correlation between the reversed metabolite levels in brain and biofluids (blood, urine and faeces) of PRE‐treated mice was analysed. As shown in Figure 4E,F, 657 significant metabolite–metabolite correlations between blood and Ccx were observed, 951 between urine and Hp and 804 between faeces and Hp (*r *> 0.80, *p <* .05) (see the Supporting Information for details). These results indicated that Hp and Ccx regions might be the most susceptible to AD, and PRE treatment had a beneficial effect by correcting the metabolic disturbance that occurred in Hp and Ccx.

**FIGURE 4:**
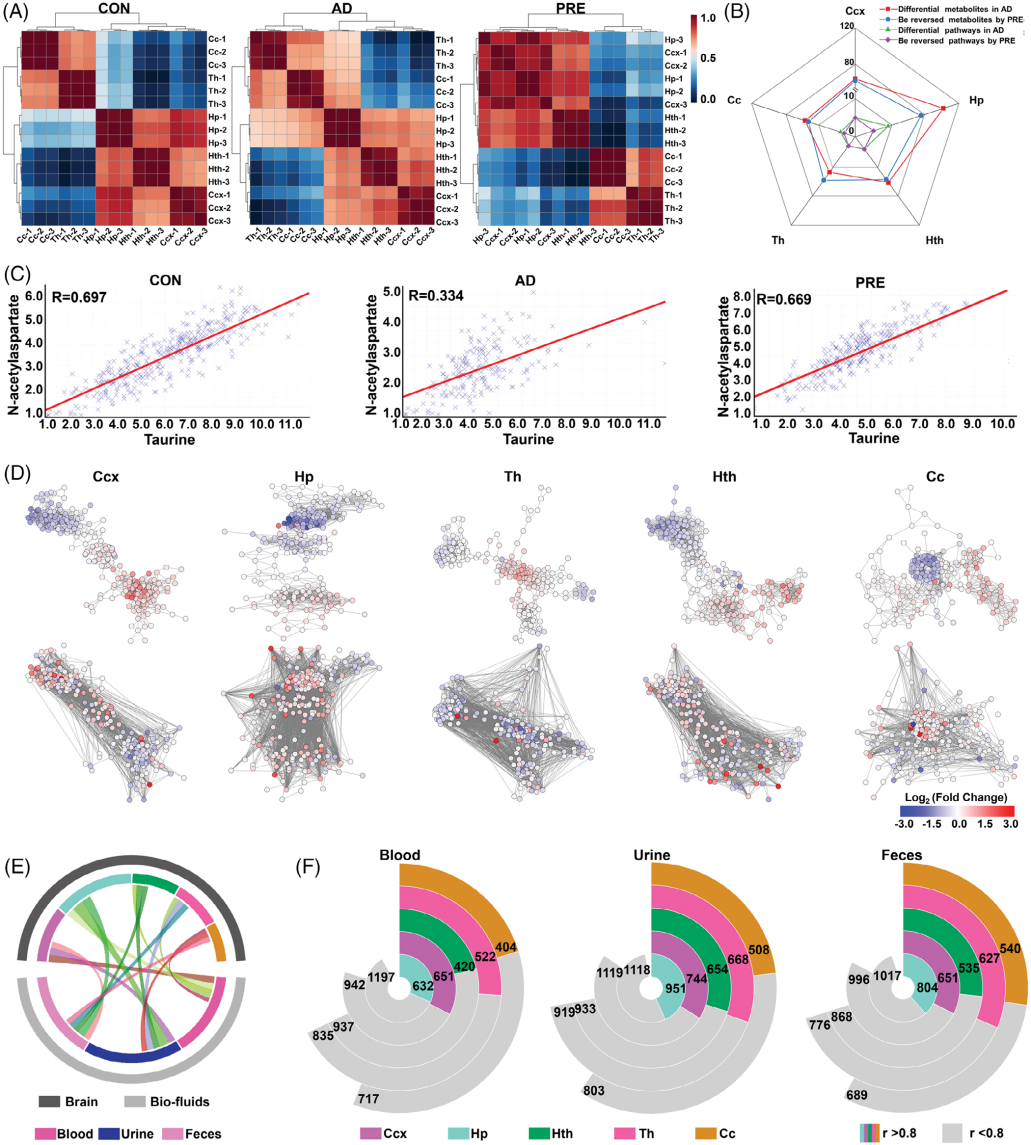
Regulatory effect of PRE on system‐wide metabolic disturbance in AD model mice revealed by integrated metabolomics analysis. (A) Correlation matrices for brain regions of CON, AD and PRE‐treated mice across five brain regions. (B) The statistical results of corrected metabolites and metabolic pathways in five brain regions of PRE‐treated mice. (C) Correlation of taurine and N‐acetylaspartate in Ccx regions from CON, AD and PRE ‐treated mice (*n* = 3per group). (D) Network visualisation of the metabolite correlations in five brain regions between AD and CON mice (top panel) and between PRE‐treated and AD mice (bottom panel). The edges are correlations of inter‐metabolite with *R* > 0.90. The nodes represent metabolites. The nodes of the network are colour‐coded based on the log_2_ (fold changes) values of metabolites in each brain region. (E) Circos plots showing the correlations of PRE‐changed differential metabolites between five brain regions and biofluids in AD model mice and (F) the statistical results of those metabolite–metabolite correlations. Ccx, cerebral cortex; Hp, hippocampus; Hth, hypothalamus; Th, thalamus; Cc, corpus callosum.

Overall, the regulatory effect of PRE on the disturbances of system‐wide metabolic homeostasis in AD mice was demonstrated by MS‐based multi‐dimensional metabolomics. Spatial metabolomics revealed that PRE treatment significantly reversed the brain region‐specific metabolic disturbances in AD mice and improve the metabolic coordination among five brain regions. Meanwhile, the perturbed metabolic profiles in the blood, urine and faeces of AD mice were significantly improved after PRE treatment, particularly concentrated in pathways associated with glycerophospholipid and sphingolipid metabolism. More importantly, PRE significantly improved the metabolic coordination between brain regions and blood, urine and faeces in AD mice, especially the inter‐metabolite correlation between blood, urine, faeces and the Hp and Ccx regions, indicating that PRE could alleviate disturbance of system‐wide metabolic homeostasis via regulating the metabolism coordination between central and peripheral system. Our findings may provide a new strategy for the multi‐targeted treatment of AD.

## AUTHOR CONTRIBUTIONS

*Conceptualisation, investigation, methodology, validation and writing—original draft*: Yanwen Chen. *Investigation, methodology and validation*: Lisha Zhao. *Methodology and validation*: Shuo Cai. *Investigation, methodology and validation*: Yuchen Zou. *Conceptualisation, investigation, methodology and validation*: Weiwei Tang. *Conceptualisation, supervision, writing—review and editing*: Bin Li. All authors reviewed the manuscript and approved the submitted version.

## CONFLICT OF INTEREST STATEMENT

The authors declare no conflicts of interest.

## FUNDING INFORMATION

This work was supported by the National Natural Science Foundation of China (No. 82374028, No. 82304894, and No. 81773873) and the Jiangsu Funding Program for Excellent Postdoctoral Talent. The content is solely the responsibility of the authors and does not necessarily represent the official views of the funding agencies.

## ETHICS STATEMENT

All animal protocols were approved by the Institutional Animal Care and Use Committee of China Pharmaceutical University, which are in accordance with the National Institutes of Health guidelines.

## Supporting information



SUPPORTING INFORMATION

## References

[1] Tournissac M , Leclerc M , Valentin‐Escalera J , et al. Metabolic determinants of Alzheimer's disease: a focus on thermoregulation. Ageing Res Rev. 2021;72. 10.1016/j.arr.2021.101462 34534683

[2] Munoz‐Moreno E , Simoes RV , Tudela R , et al. Spatio‐temporal metabolic rewiring in the brain of TgF344‐AD rat model of Alzheimer's disease. Sci Rep. 2022;12:16958. 36216838 10.1038/s41598-022-20962-6 PMC9550832

[3] Graham SF , Holscher C , McClean P , et al. 1H NMR metabolomics investigation of an Alzheimer's disease (AD) mouse model pinpoints important biochemical disturbances in brain and plasma. Metabolomics. 2013;9:974‐983.

[4] Zeng W , Wu AG , Zhou XG , et al. Saponins isolated from Radix polygalae extent lifespan by modulating complement C3 and gut microbiota. Pharmacol Res. 2021;170:105697. 34062240 10.1016/j.phrs.2021.105697

[5] Zhao X , Cui Y , Wu P , et al. Polygalae Radix: a review of its traditional uses, phytochemistry, pharmacology, toxicology, and pharmacokinetics. Fitoterapia. 2020;147:104759. 33069838 10.1016/j.fitote.2020.104759

