# Supplementary material for: MS‐based multi‐dimensional metabolomics reveals protective effect of Polygalae Radix against metabolic disturbances in Alzheimer's disease mice
Source: Clin Transl Med. 2025 Apr 15;15(4):e70292. doi: 10.1002/ctm2.70292 (PMC12000232; doi:10.1002/ctm2.70292)
Supplement: Supplementary file 1 — SUPPORTING INFORMATION [file CTM2-15-e70292-s001.pdf]

Supporting Information

MS-based Multi-dimensional Metabolomics Reveals Protective Effect of Polygalae Radix Against Metabolic Disturbances in Alzheimer's Disease Mice

Yanwen Chen, Lisha Zhao, Yuchen Zou, Weiwei Tang*, Bin Li*

State Key Laboratory of Natural Medicines and School of Traditional Chinese Pharmacy, China Pharmaceutical University, No. 639 Longmian Avenue, Nanjing, Jiangsu Province, 210009, China

* Corresponding authors:

E-mail: weiweitang@cpu.edu.cn (Weiwei Tang), binli@cpu.edu.cn (Bin Li)

Figure S1. Total ion chromatograms of PRE based on UPLC-Q/TOF-MS.

Figure S2. Pharmacological effects of PRE on the improvement of learning and memory impairment in the AD model mice.

Figure S3. PRE-treated improves the pathological characteristics in the AD mice brain.

Figure S4. PCA and PLS-DA score plot of Hp, Th, Hth, and Cc regions based on MALDI-MS.

Figure S5. Volcano plot displayed differential metabolites with fold-change >1.5 in the Hp, Th, Hth, and Cc regions between AD and CON groups, as well as PRE and AD groups.

Figure S6. Heat map (left) and ion images (right) of differential metabolites by PRE reverses in Hp and Th regions of AD mice.

Figure S7. Heat map (left) and ion images (right) of differential metabolites by PRE reverses in Hth and Cc regions of AD mice.

Figure S8. The enriched metabolic pathways for the differential metabolites in the Hp and Th regions of AD model mice and PRE-treated mice.

Figure S9. The enriched metabolic pathways for the differential metabolites in the Hth and Cc regions of AD model mice and PRE-treated mice.

Figure S10. PCA, PLS-DA, and OPLS-DA score plot of urine and faeces based on UPLC Q/TOF MS.

Figure S11. PRE ameliorates the metabolic perturbation in the blood of AD mice.

Figure S12. Volcano plot displayed differential metabolites with fold-change >1.5 in urine and faeces between AD and CON groups, as well as PRE and AD groups.

Figure S13. Heat map of differential metabolites by PRE reverses in the urine and faeces of AD mice.

Figure S14. The enriched metabolic pathways for the differential metabolites in the urine and faeces of AD model mice and PRE-treated mice.

Figure S15. Correlation matrices of differential metabolites in 5 brain regions of three groups of mice.

Figure S16. Correlation of taurine and N-acetylaspartate in the Hp region and the whole brain of three groups of mice.

Figure S17. The changing trends of taurine and arachidonic acid in three groups.

Figure S18. PRE-treated improves the neuroinflammation in the AD mice brain.

Table S1. Characterisation of chemical constituents in PRE

Table S2. The regulated differential metabolites observed in Ccx region of PRE-treated AD mice

Table S3 The regulated differential metabolites observed in Hp region of PRE-treated AD mice

Table S4 The regulated differential metabolites observed in Th region of PRE-treated AD mice

Table S5 The regulated differential metabolites observed in Hth region of PRE-treated AD mice

Table S6 The regulated differential metabolites observed in Cc region of PRE-treated AD mice

Table S7 The regulated differential metabolites observed in blood of PRE-treated AD mice

Table S8 The regulated differential metabolites observed in urine of PRE-treated AD mice

Table S9 The regulated differential metabolites observed in faeces of PRE-treated AD mice

Materials and Methods

Chemicals and Reagents

HPLC-grade methanol (MeOH) and acetonitrile (ACN) were purchased from Merck (Muskegon, MI). HPLC-grade trifluoroacetic acid (TFA) and formic acid (FA) were purchased from Sigma-Aldrich (St. Louis, MO). Ketoprofen, (S)-2-amino-3-(2-chlorophenyl) propanoic acid, ammonia hydroxide (NH_4OH), hydrochloric acid (HCl), potassium chloride (KCl), sodium borohydride ($NaBH_4$), potassium permanganate ($KMnO_4$), oxalic acid, and ethanol were purchased from Aladin (Shanghai, China). 2,5-dihydroxybenzoic acid (DHB), 1,5-naphthalenediamine (1,5-DAN), thioflavin S, and donepezil hydrochloride (DP) were purchased from Sigma-Aldrich (St. Louis, MO). 4-Aminocinnoline-3-carboxamide (4-AC) was purchased from Bidepharm (Shanghai, China). Deionized water was generated by using a Millipore purification system (Millipore, Billerica, MA). The ELISA Assay Kits were purchased from Shanghai Enzyme-linked (Shanghai, China). PR extract (PRE) was self-made, and its chemical composition was characterised by UHPLC-MS/MS (**Figure S1** and **Table S1**).

Animal Study

Male 30-week-old APPswe/PSEN1dE9 (APP/PS1) double transgenic mice (28-32g) were purchased from CAVENS (Changzhou, China) (License: SCXK 2016-0010), and kept in an environmentally controlled breeding room (12 h dark/light cycle at 24 °C with humidity of 53%) for at least 1 week before the experiment. 30 AD model mice were randomly divided into 3 groups (n=10 per group): i) AD model group was orally treated with 0.5% sodium carboxymethyl cellulose (CMC-Na) for 26 weeks, ii) DP group was orally treated with DP (2 mg/kg body weight per day) for 26 weeks, and iii) PRE group was orally treated with PRE (50 mg/kg body weight per day) for 26 weeks. 10 male 30-week-old C57BL/6J mice (28-32 g) were treated with 0.5% CMC-Na as a control group (CON). Animal experiments were conducted by the Guidelines for Animal Experimentation of China Pharmaceutical University (Nanjing, China) and approved by the Animal Ethics Committee of this institution.

Behavioral Experiments

After continuous treatment for 26 weeks, the Morris water maze (MWM) test and new object recognition (NOR) test were performed to evaluate the spatial learning and memory ability of AD mice as previously described.[1-3] All tests were performed in blocks of 4-5 tests and only on a selected number of mice to avoid overstressing the animals. Each rater was blinded to the test results of the other four raters.

Morris Water Maze (MWM) Test

After continuous treatment for 26 weeks, the MWM test was performed to detect the spatial learning and memory ability as previously described. Briefly, the WMW test apparatus is composed of a 120 cm diameter, 50 cm tall circular water tank and a 10 cm diameter, 30 cm

tall circular platform, and the surface of all components is white. In the WMW acquisition phase, the cylindrical platform was fixedly placed in the middle of the first quadrant of the tank, and 0.5-1.0 cm under the water to ensure unseen by mice. The mice were placed into the tank at water level facing the tank wall, and allowed to find the hidden platform within 90 s. The time spent to find and climb onto the platform was recorded as escape latency. If the mice failed to reach the platform within 90 s, they were guided to the platform, and the escape latency was recorded as 90 s. All the mice were trained 3 times a day for 6 consecutive days. After climbing the platform, they were allowed to remain on it for 10 s to remember the site of the platform.

After the WMW acquisition test, the WMW probe test was operated to evaluate the spatial memory retention of these mice. The mice were placed into the tank while the platform was removed, and then the exploration time and distance in the target quadrant, the number of platform-site crossovers, and the swimming speed were recorded within 60 s.

New Object Recognition (NOR) Test

The NOR test, another behavioral assay that is commonly used for the investigation of learning and memory in mice, was performed as previously described. In short, the NOR proceeded in a square open field apparatus and included three sessions: habituation session, training session, and testing session. Two white cylinders with a height of 10 cm and a diameter of 8 cm, and one black cube with a side length of 8 cm, but have the same degree of complexity and are made of non-breakable material, were used as the objects to be recognised. In the habituation session, gently take the mice from their cage into the empty open field, and keep the mouse heads facing the wall. Each mouse was allowed to freely explore the open field for 5 min. At the end of the habituation session, the mouse was placed in a new empty cage. Before the next mouse was placed into the open field, thoroughly cleaned the apparatus using 70% vol/vol ethanol and air-dried it. In the training session, place the two white cylinders in the two nearby corners of the open field, and 5 cm away from the nearby walls. 24 h after habituation, gently take the mouse from its cage into the open field, keep the mouse head facing the wall, and equidistant from the 2 identical objects. Each mouse was allowed to freely explore the open field and the objects for 10 min. At the end of the training session, the mouse was removed and placed in a new empty cage. Before the next mouse was trained, thoroughly cleaned the apparatus and the objects using 70% vol/vol ethanol and air-dried it. After all of the mice completed the training session, one of the familiar objects was replaced by the black cube as the novel object. 24 h after training, gently take the mouse from its cage into the open field and allow the mouse to freely explore the open field for 10 min. Record the exploration time on the familiar object and the novel object of each mouse. The discrimination index (DI) of the novel object was calculated as follows: DI = (exploration time of the novel object − exploration time of the familiar object)/(exploration time of the novel object + exploration time of the familiar object).

Sample Collection

After behavioral tests, urine and faeces were collected. The urine was centrifuged at 4,500 ×g for 10 min, and the supernatant of the urine was transferred to a clean tube. Approximately 500 μL of blood was collected from the orbital sinus of the mouse. After standing for 2 h at room temperature, the whole blood was centrifuged at 4,500 ×g for 10 min, and the supernatant of serum was transferred to a clean tube. Next, the mouse brains were quickly collected over ice, washed with ice-cold saline to remove blood taint, and wiped with filter paper to get rid of saline. For the biochemical assay, the cerebral cortex and hippocampus tissue were collected into a pre-weighed Eppendorf tube. All samples were kept at -80 °C until use.

Sample Preparation for LC-MS

Took 100 μL of serum and urine supernatants, respectively, and added 400 μL of precooled MeOH-ACN (1:1, V/V) containing two internal standards (IS), ketoprofen (0.4 μg/mL) and 2-chloro-L-phenylalanine (1.0 μg/mL). After being thoroughly mixed by vortex, the mixture was centrifuged at 16,000 ×g for 10 min. Took 400 μL supernatants of all samples, evaporated the solvent, added 100 μL of precooled ACN-H_2O (3:7, V/V) to the residue, and mixed on a vortex mixer for 3 min. The mixture was centrifuged at 16,000 ×g for 10 min, and the supernatant was collected for LC-MS analysis (see the Supporting Information for details on LC-MS experiments).

The faeces were freeze-dried and ground into a fine powder. Accurately weighed 50 mg and transferred into a centrifuge tube. 500 μL of precooled deionized water was added, mixed thoroughly on a vortex mixer, and ultrasonically extracted for 40 min at 4 °C. Centrifuged at 16,000 ×g for 10 min at 4 °C, and took 200 μL of the supernatant. Similarly, 500 μL of precooled MeOH was added to the residue and treated in the same way as water extracts. 200 μL of MeOH extracts were mixed with the same volume of water extracts. Finally, 400 μL of precooled MeOH-ACN containing two IS were added to 200 μL of the combined extracts, and the same procedure was followed as serum and urine sample preparation.

LC-MS/MS Experiments

The metabolic profiles of CON mice, AD mice, and PRE-treated AD mice were performed using UPLC-ESI-MS/MS. 50 μL prepared bio-fluids (serum, urine, and faeces) samples were mixed with 400 μL MeOH: ACN (1:1, v/v), vortexed for 5 min, and centrifuged at 16,000 ×g for 10 min at 4 °C. After dried by nitrogen (N_2) blowing, 50 μL ACN: H_2O (1:1, v/v) was added to re-dissolve the residuals. The supernatant was injected into the LC−MS/MS system for analysis. LC−MS/MS analysis was performed using an Agilent 6530 Q-TOF mass spectrometer connected to an Agilent 1260 series UHPLC system (Agilent Technologies, CA). Analytes were separated on an Agilent Proshell 120 C18 column (4.6 mm × 50 mm, 1.8 μm) preceded by a guard column (5 mm × 4.6 mm, 1.8 μm, Agilent Eclipse XDB-C18) at 30 °C. Mobile phase A was water containing 0.1% formic acid (v/v), and phase B was acetonitrile. The flow rate of the mobile phase was 0.4 mL/min. The gradient program was conducted as follows: 0-2 min, 10-

40% B; 2-4 min, 40-50% B; 4-6 min, 50-60% B; 6-9 min, 60% B; 9-12 min, 60-90% B; 12-14 min, 90-100% B; and 14-15 min, 100% B. The injection volume was 2 μL. Samples were analysed using the electrospray ionisation (ESI) source in both positive and negative modes, respectively. The capillary voltage for positive and negative modes was +4.0 kV and −3.5 kV, respectively. The other source parameters were as follows: drying gas temperature, 350 °C; drying gas flow, 10.0 L/min; sheath gas temperature, 300 °C; sheath gas flow, 11 L/min; nebulizer pressure, 45 psi.

Brain Tissue Preparation for MALDI-MS Imaging

In all cases, 12 μm-thick mouse brain coronal sections were obtained at -20 °C using a cryostat (Leica, Germany), and thaw-mounted onto ITO glass slides for MALDI-MS imaging. Before matrix coating, the brain sections were placed in a vacuum desiccator to dehydrate for 10 min at room temperature. A laboratory-constructed electro-sprayer was used to uniformly apply matrix solutions on brain sections. Depending on the analytes to be detected, 200 μL of 30 mg/mL DHB in MeOH-H_2O (4:1, V/V) containing 0.5 % TFA, or 200 μL of 5 mg/mL 1,5-DAN in EtOH-H_2O (1:1, V/V) containing 0.5 % HCl, or 500 μL of 3 mg/mL 4-AC in ACN-H_2O (4:1, V/V) containing 1.0 % TFA or 1.0 % NH_4OH were deposited on brain tissue sections for MALDI-MSI in the positive (DHB•TFA and 4-AC•TFA) and negative (1,5-DAN•HCl and 4-AC•NH_4OH) ion mode, respectively.

Brain Tissue Preparation for ELISA

The cerebral cortex and hippocampus tissue were homogenised in ice-cold PBS buffer solution (0.01 M, pH=7.4) (1:10, W/V). After being centrifuged at 4,500 ×g for 10 min at 4 °C, the supernatants were collected and used for ELISA assay to estimate the level of BDNF, ROS, TNF-α, IL-6, IL-1, Ach, $A\beta_{1\text{-}42}$, and the activity of BACE1.

Thioflavin S Fluorescence Assay

Thioflavin S fluorescence assay was performed to detect the distribution and level of amyloids in mouse brains as previously described [3]. Briefly, (1) 10 μm thick mouse brain coronal sections were fixed in 4% paraformaldehyde for 12 h at room temperature; (2) rinsed with fresh deionized water (5 min, ×3); (3) incubated in 0.3% $KMnO_4$ solution for 3 min; (4) rinsed with fresh deionized water (5 min, ×3); (5) incubated in 1.0% oxalic acid until the brown color disappeared completely; (6) incubated in 1.0 % $NaBH_4$ solution for 5 min; (7) rinsed with PBS buffer solution (0.01 M, pH=7.4) for 5 min and then deionized water for 3 s, incubated in 0.3% Thioflavin S ethanol-water (1:1, V/V) solution for 10 min in the dark; (8) rinsed with 50 % ethanol (10 s, ×3), dry in the dark. The brain sections were imaged using a batch slide scanner (NanoZoomer 2.0 RS, Hamamatsu).

MALDI-MS and MSI Experiments

MALDI-MS and MSI were performed using an UltrafleXtreme MALDI TOF/TOF MS (Bruker Daltonics) with a frequency tripled Nd: YAG solid-state laser (λ=355 nm). The laser was set to the "Ultra" footprint setting at a ~100 μm diameter. Mass spectrometer calibration was performed using DHB matrix ions and a Peptide Calibration Standard Kit II (Bruker Daltonics). Tissue sections were analysed respectively in positive and negative reflectron ion mode with 100 laser shots fired at 1000 Hz and imaged with a 200 μm step size. MS data were analysed using flexAnalysis 3.4 and flexImaging 4.1 (Bruker Daltonics). All spectra were baseline-subtracted. A high-resolution MALDI timsTOF flex (Bruker Daltonics) was used for accurate *m/z* measurement. Putative identification of signals detected in the MALDI-MS was performed using *in situ* tandem MS (MS/MS) with MALDI LIFT-TOF/TOF MS and online metabolite annotation via accurate *m/z* matching to the HMDB, Lipid Maps, and METLIN databases.

Data Analysis

The imaging data set was exported to the imzML format by flexImaging 4.1 and imported into MSiReader v1.02 for data analysis or converted to the SCiLS SL file format by SCiLS Lab and imported into SCiLS Lab for data analysis (SCiLS GmbH, Bremen, Germany). The statistical analysis was performed using SCiLS Lab, Origin 9.7 (Northampton, Massachusetts, USA), and Mateboanalyst 5.0 online (www.metaboanalyst.ca). KEGG Pathway Database (www.genome.jp) was used for metabolic pathway enrichment analysis. The difference between groups was determined using One-way ANOVA followed by the Tukey multiple comparison test.

Results

UHPLC-MS/MS Profile of PRE and the Pharmacological Experiment Results of PRE Anti-AD

The chemical composition of PRE was characterised by UHPLC-MS/MS, and the total ion chromatogram (TIC) is shown in **Figure S1**. A total of 60 ingredients in PRE were identified and determined based on the accurate molecular weight provided by primary mass spectrometry and fragment ion information provided by secondary mass spectrometry referenced by published reports, public databases, and standard references. These ingredients mainly include 29 saponins, 25 oligosaccharide esters, and 6 Xanthones, and their identification information is listed in **Table S1**.

WMW and NOR tests were first performed to assess the improvement of spatial learning and memory function in 57-week-old AD model mice after 26 weeks of PRE treatments (**Figure S2A**). The orientation navigation experiment showed that the AD model mice displayed significantly longer escape latency than the CON mice on days 2-6, indicating that spatial memory deficits occurred in AD model mice. However, the behavioral performance of AD model mice was significantly improved in PRE- and DP-treated (positive control) groups after a 6-day learning period. (**Figure S2B**). Additionally, a spatial probe test was conducted on the

7th day of the MWM test to evaluate spatial reference memory. The AD model mice showed a significant decrease in exploration time, platform crossing numbers, and exploration distance in the target quadrant as compared with the CON mice, however, these could be substantially ameliorated after 26 weeks of oral administration of PRE (**Figure S2C-F**). The average swim speed was not significantly different among the four groups (**Figure S2G**), indicating that these behavioral divergences in the MWM test were not attributed to individual physical strength. In the NOR test, the representative motion tracks of different groups are shown in **Figure S2H**. On day 3, the AD model mice spent less time exploring the novel object and had a lower discrimination index than CON mice (**Figure S2I-J**). In contrast, the PRE-treated AD model mice showed a significant preference for the novel object, indicating a clear improvement in recognition memory. Furthermore, the results of biochemical tests showed that PRE treatment could remarkably reduce the levels of Aβ plaques (**Figure S2K-L**) and $A\beta_{1\text{-}42}$ monomers, and increase the levels of acetylcholine (Ach) and brain-derived neurotrophic factor (BDNF) in the cerebral cortex (Ccx) and hippocampus (Hp) of AD model mice (**Figure S3**). The results of behavioral and pharmacological experiments indicated that the impairment of learning and memory in AD model mice could be ameliorated by chronic oral administration of PRE. On the other hand, DP-treated mice performed well in behavioral experiments, and a significant increase in Ach levels was observed in the Ccx and Hp regions (**Figure S2B-J** and **Figure S3C**). However, the treatment with DP did not have any influence on the levels of Aβ plaques, $A\beta_{1\text{-}42}$ monomer, and BDNF (**Figure S2K-L** and **Figure S3**), which is consistent with DP's role as a cholinesterase inhibitor.[4]

PRE-treatment Ameliorates the System-wide Metabolic Homeostasis in AD Mouse

To access the system-wide metabolic homeostasis, the correlation between the reversed metabolite levels in brain tissues and biofluids of PRE-treated mice was analysed. As shown in **Figure 4E-F**, 657 significant metabolite–metabolite correlations between blood and Ccx were observed, 632 between blood and Hp, 951 between urine and Hp, 774 between urine and Ccx, 804 between faeces and Hp, and 651 between faeces and Ccx (r>0.80, p<0.05). These results indicated that Hp and Ccx regions might be the most susceptible to AD, and PRE treatment had a beneficial effect by correcting the metabolic disturbance that occurred in Hp and Ccx. For example, the level of taurine significantly increased in the faeces of AD model mice but severely decreased in Hp and Ccx. These alterations changed after PRE treatment, and the trend displayed a highly negative correlation (**Figure S17A**). Taking arachidonic acid as another example, the alteration trend of arachidonic acid in Hp and Ccx was highly consistent with that in blood, indicating that PRE might regulate the distribution balance of arachidonic acid between the brain and biofluids (**Figure S17B**).

Correlation Analysis Between Metabolites and Neuroinflammatory Factors in the Ccx and Hp regions

Multiple epidemiological, clinical, and neuropathological studies have consistently demonstrated the presence of inflammation in the vulnerable regions of the AD brain, such as Ccx and Hp.[5,6] As shown in **Figure S18A**, PRE treatment significantly reduced IL-1, IL-6, and reactive oxygen species (ROS) levels, and slightly decreased tumor necrosis factor (TNF)-α levels in the brains of AD model mice. Pearson correlation analysis revealed a significant positive correlation between the levels of IL-1, IL-6, and TNF-α and two metabolites PC(38:6) and arachidonic acid in both Hp and Ccx regions. Additionally, the significant positive correlation of psychosine sulfate with IL-6 and TNF-α was only observed in Ccx, and phosphodimethylethanolamine, 2,5-dihydrophenylalanine, and AMP with IL-6 only in Hp (p<0.01). However, the levels of taurine, GABA, L-carnitine, citric acid, and 4-guanidinobutanoic acid in both Hp and Ccx were significantly negatively correlated to those of inflammatory factors (p<0.01). On the other hand, ROS showed a significant negative correlation with taurine, GABA, L-carnitine, citric acid, and 4-guanidinobutanoic acid, but a significant positive correlation with PC(38:6) and AA in the Ccx of PRE-treated mice (p<0.01). In Hp, ROS was negatively correlated with glycerophosphocholine, citric acid, and N-acetylaspartate (**Figure S18B**). Previous studies have demonstrated the anti-aging and antioxidant effects of taurine,[7] as well as the proinflammatory effects of arachidonic acid and PCs.[8,9] Therefore, PRE could alleviate inflammation and oxidative stress responses in Hp and Ccx of AD mice via the regulation of brain region-specific metabolic disorders.

Limitation

While our study has made significant strides in establishing the cause-effect between the anti-AD effect of PRE and its regulation of metabolic disturbances in the brain and biofluids, and illustrating the pivotal role of PRE in alleviating system-wide metabolic disturbances in AD model mice by regulating metabolic coordination between different regions in brain as well as between the brain and periphery, further research is essential to identify and validate the key metabolites and functional enzymes that PRE regulates in these metabolic disturbances. However, the sample size for spatial metabolomics analysis was limited by cost and time constraints, as well as the technical capabilities of MALDI-MSI. Moreover, the current study was conducted exclusively on male cohorts. Furthermore, the roles and mechanisms of key metabolites, such as taurine and N-acetylaspartate, in PRE-mediated regulation of system metabolic disorders in AD need further verification. Finally, this study focuses on how PRE alleviates AD by regulating metabolic disorders, further research is needed to identify the specific components in PRE that contribute to its anti-AD effects.

Figure S1

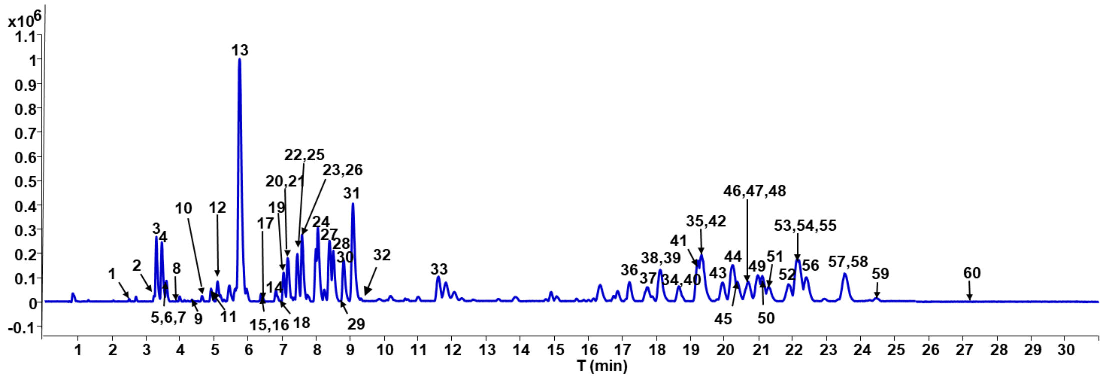

Figure S1. Total ion chromatograms of PRE obtained with UPLC-Q/TOF-MS in the positive ion mode. See **Table S1** for metabolite identification.

Figure S2

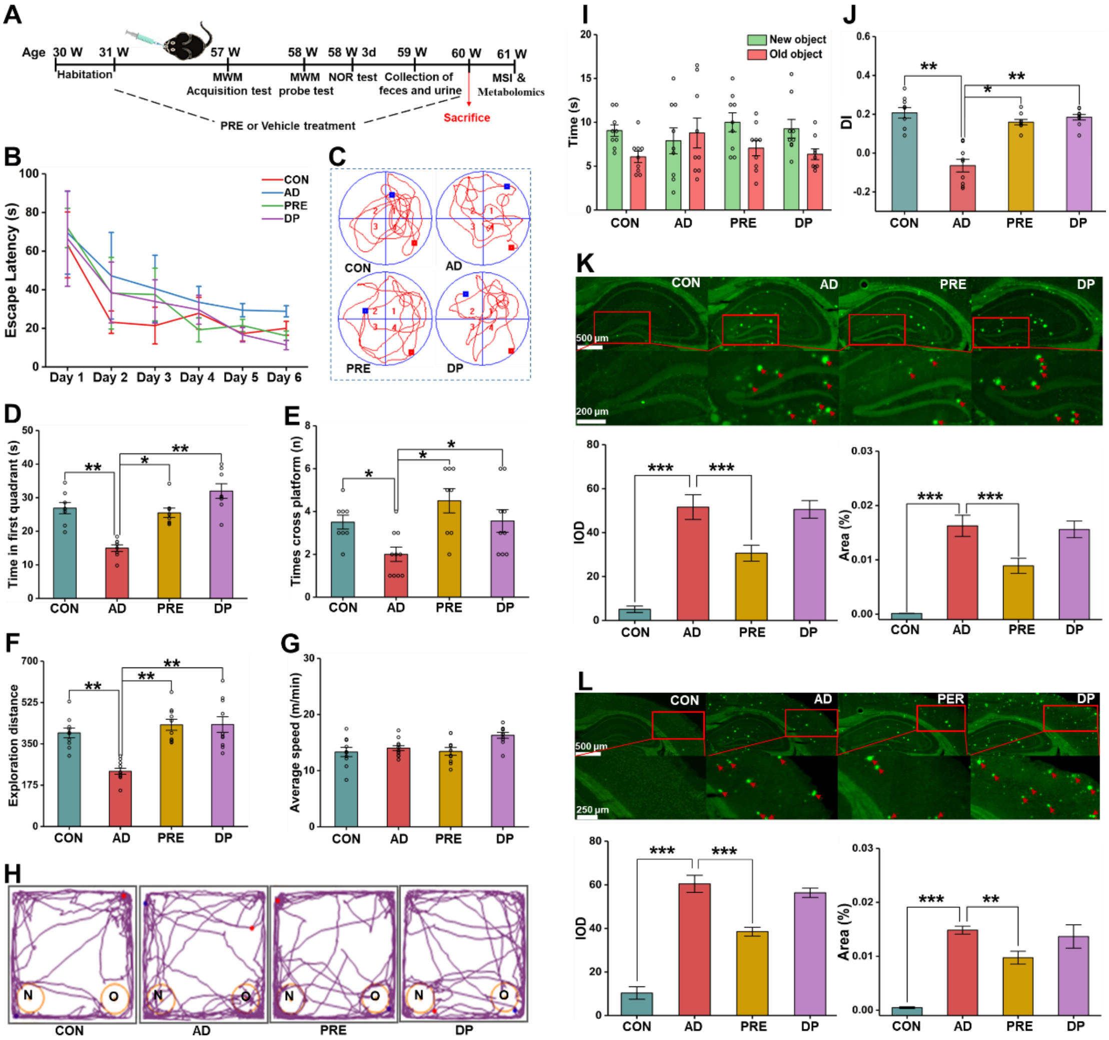

Figure S2. Pharmacological effects of PRE on the improvement of learning and memory impairment in the AD model mice. (A) The scheme of animal experiments. (B) The escape latency in the place navigation phase of the Morris water maze (MWM) test. (C) Representative motion tracks, (D) exploration time in the target quadrant, (E) times across the platform, (F) exploration distance in the target quadrant, and (G) average swimming speed of each group of mice in the spatial probe phase. (H) Representative motion tracks, (I) exploration time, and (J) the discrimination index in the new object recognition (NOR) test. (K-L) Representative thioflavin-S-stained sections of Ccx and Hp with respective statistical analysis results of IOD and area of Aβ plaques. All data are presented as the mean ± SEM (n=10 mice per group in MWM and NOR test, n=3 mice per group in Figure 1K and L). **p*<0.05, ** *p*<0.01, *** *p*<0.001, by One-way ANOVA test. Ccx, cerebral cortex; Hp, hippocampus; AD, AD model group; CON, control group; DP, donepezil hydrochloride treatment group; PRE, PRE treatment group; DI, discrimination index; IOD, integrated optical density; MSI, mass spectrometry imaging.

Figure S3

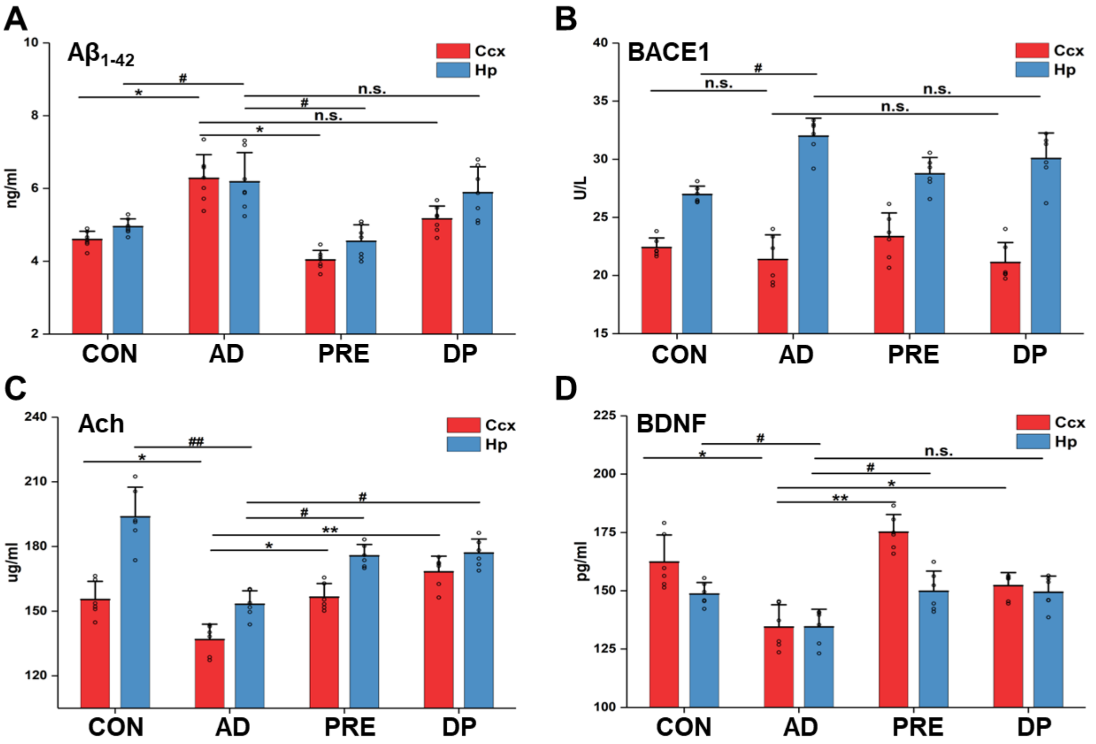

Figure S3. PRE-treated improves the pathological characteristics in the AD mice brain. (**A-D**) The levels of Aβ1-42, BACE1, Ach, and BDNF in Ccx and Hp detected by ELISA. # p<0.05, ## p<0.01, * p < 0.05, ** p < 0.01, and n.s. indicates no significant difference, by the One-way ANOVA test. Ccx, cerebral cortex; Hp, hippocampus.

Figure S4

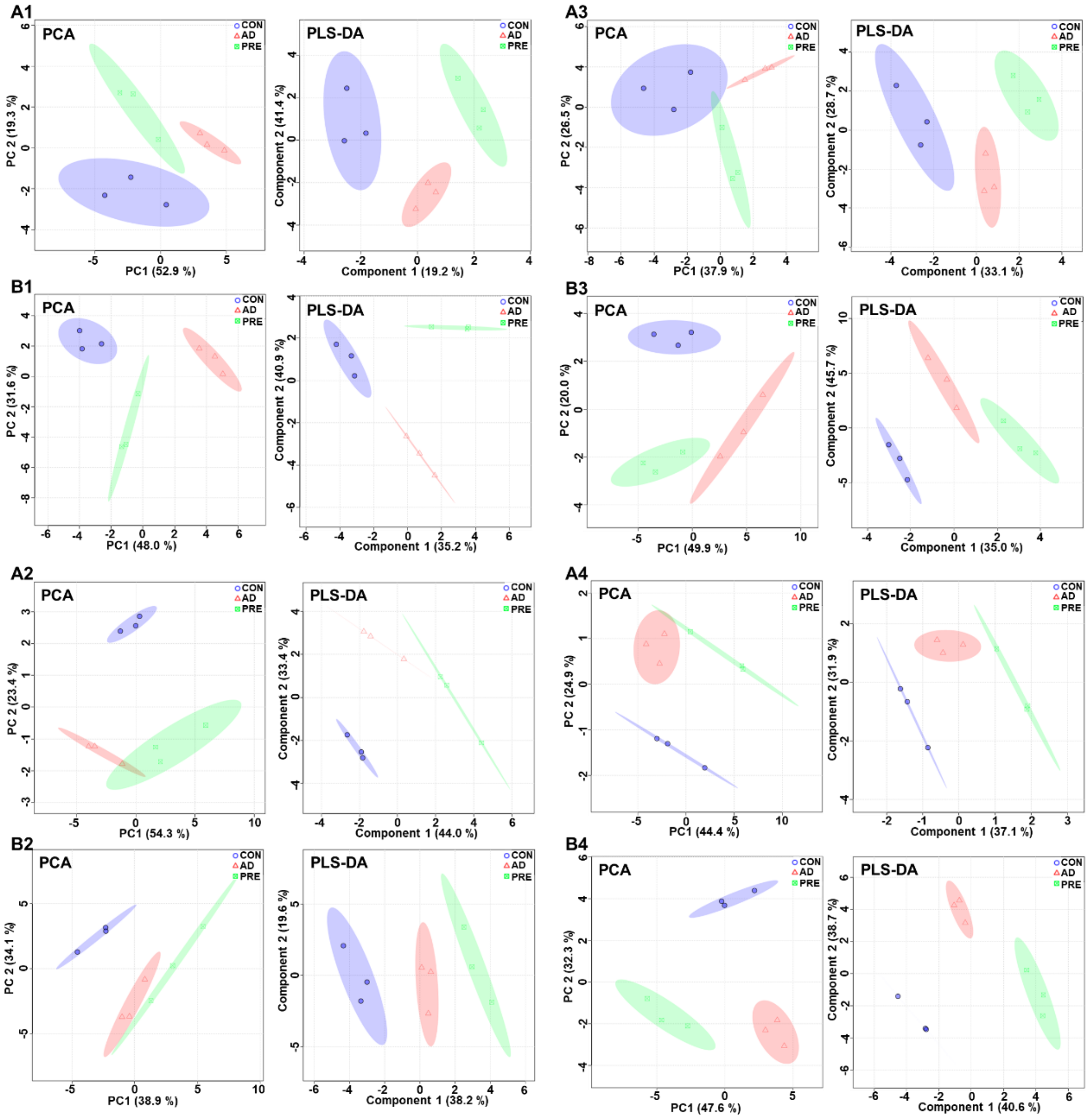

Figure S4. PCA and PLS-DA score plot of (**A1** and **B1**) Hp, (**A2** and **B2**) Th, (**A3** and **B3**) Hth, and (**A4** and **B4**) Cc regions based on MALDI-MS in negative (**A1-A4**) and positive (**B1-B4**) ion mode.

Figure S5

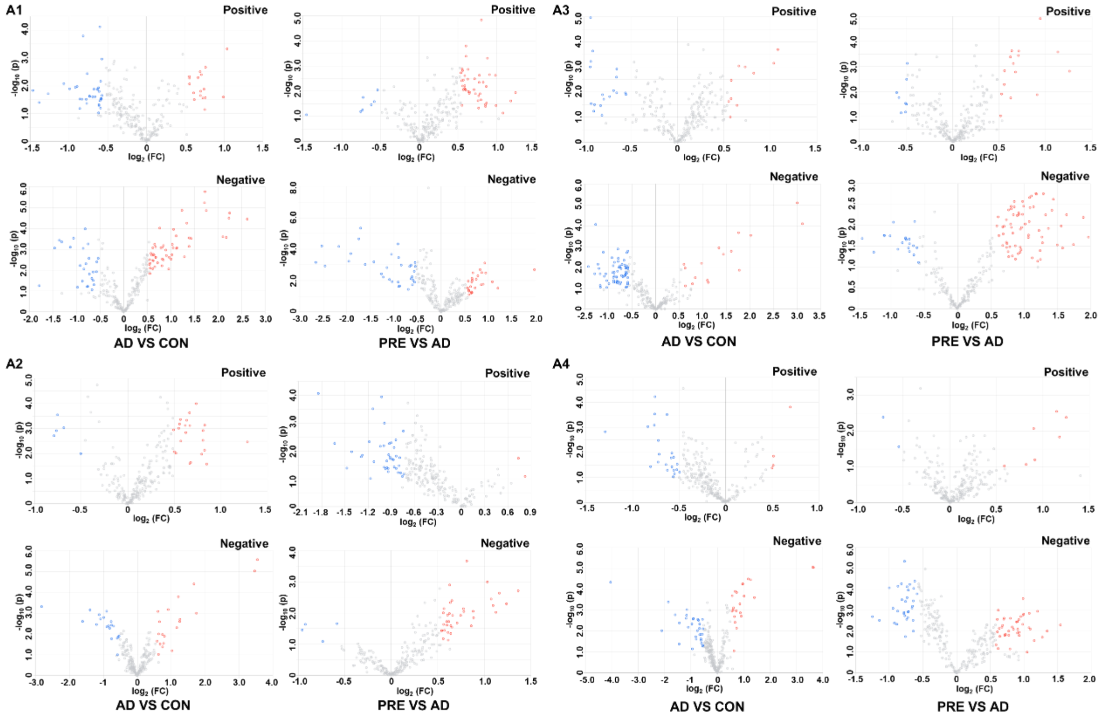

Figure S5. Volcano plot displayed differential metabolites with fold-change >1.5 in (**A1**) Hp, (**A2**) Th, (**A3**) Hth, and (**A4**) Cc regions between AD and CON groups, as well as PRE and AD groups.

Figure S6

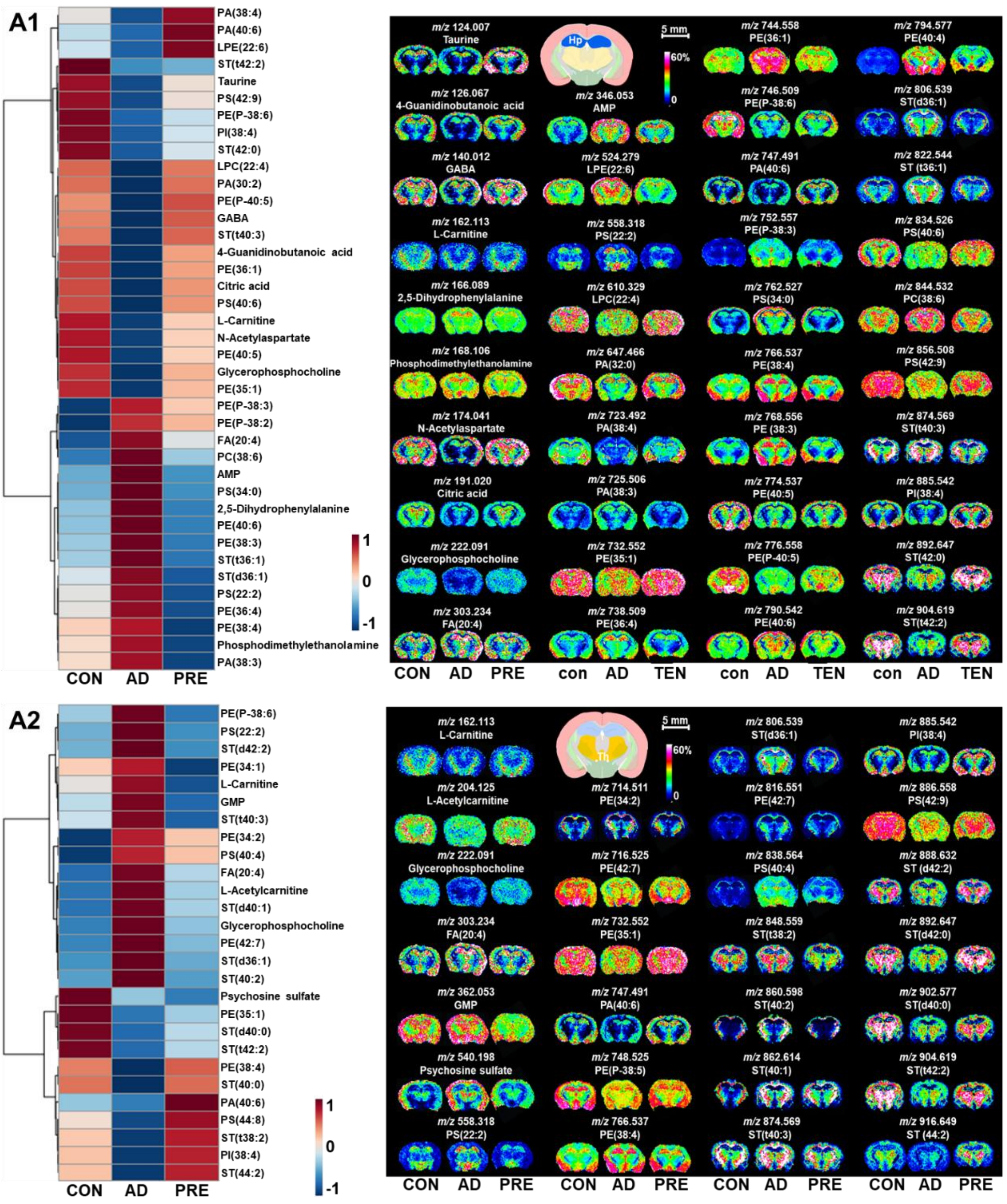

Figure S6. Heat map (left) and ion images (right) of differential metabolites by PRE reverses in Hp (**A1**) and Th (**A2**) regions of AD mice.

Figure S7

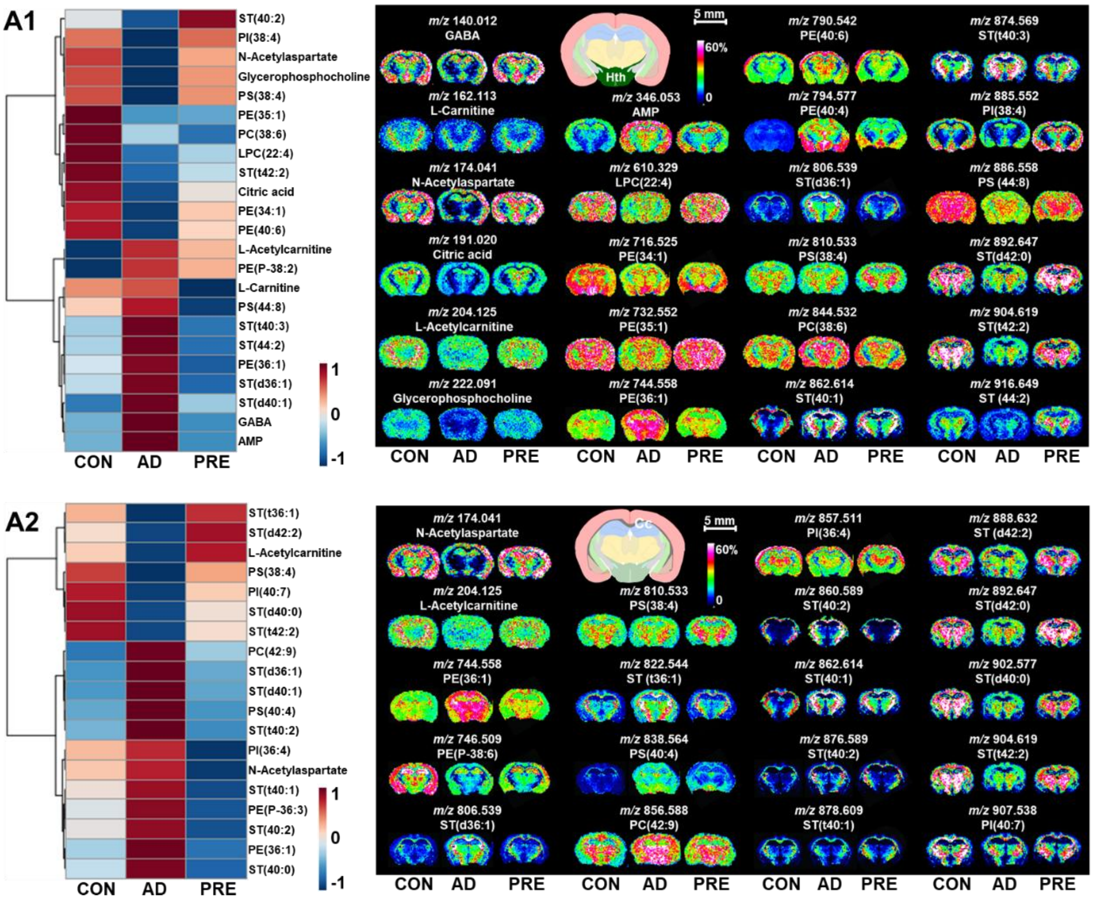

Figure S7. Heat map (left) and ion images (right) of differential metabolites by PRE reverses in the Hth (**A1**) and Cc (**A2**) regions of AD mice.

Figure S8

A1

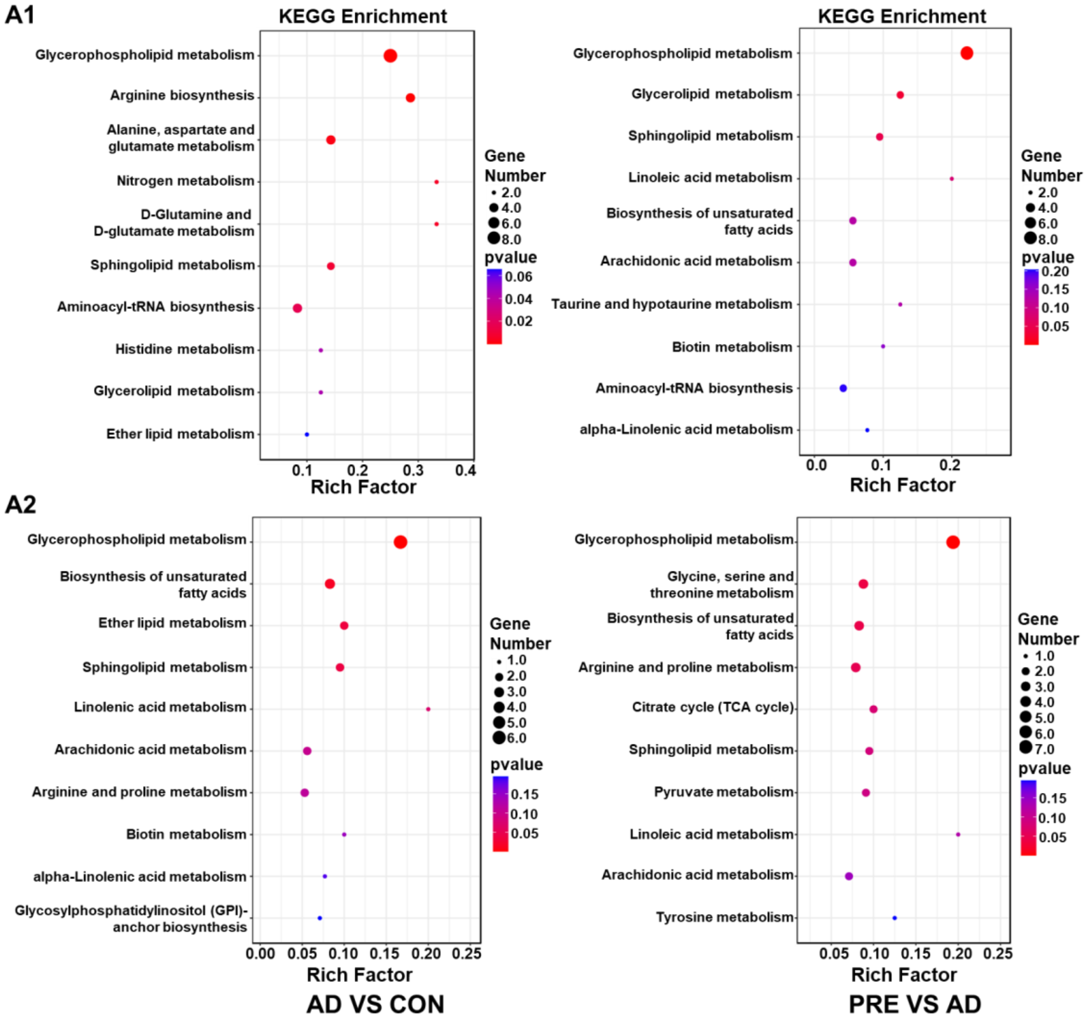

Figure S8. The enriched metabolic pathways for the differential metabolites in Hp (**A1**) and Th (**A2**) regions of AD model mice and PRE-treated mice.

Figure S9

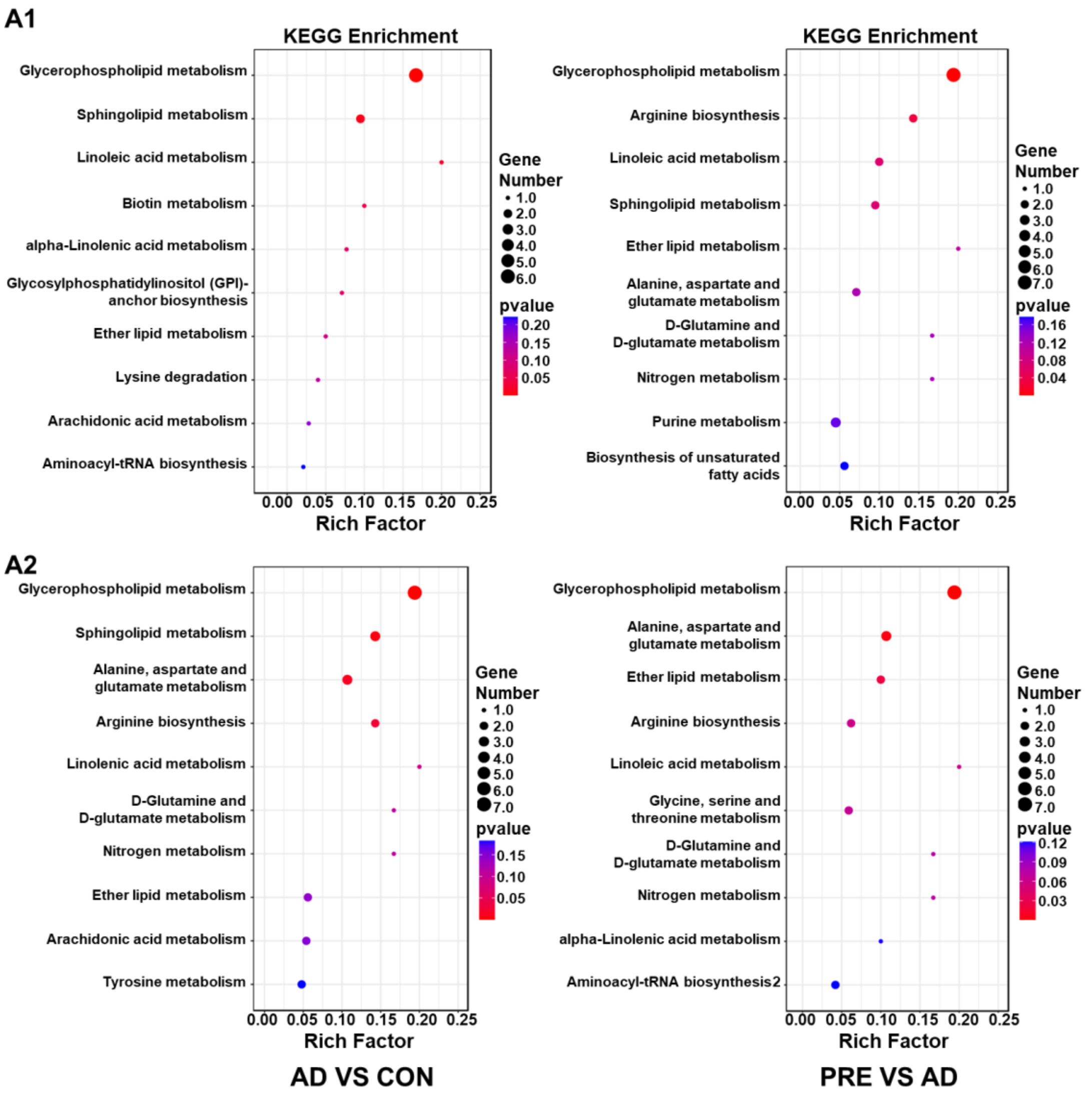

Figure S9. The enriched metabolic pathways for the differential metabolites in Hth (**A1**) and Cc (**A2**) regions of AD model mice and PRE-treated mice.

Figure S10

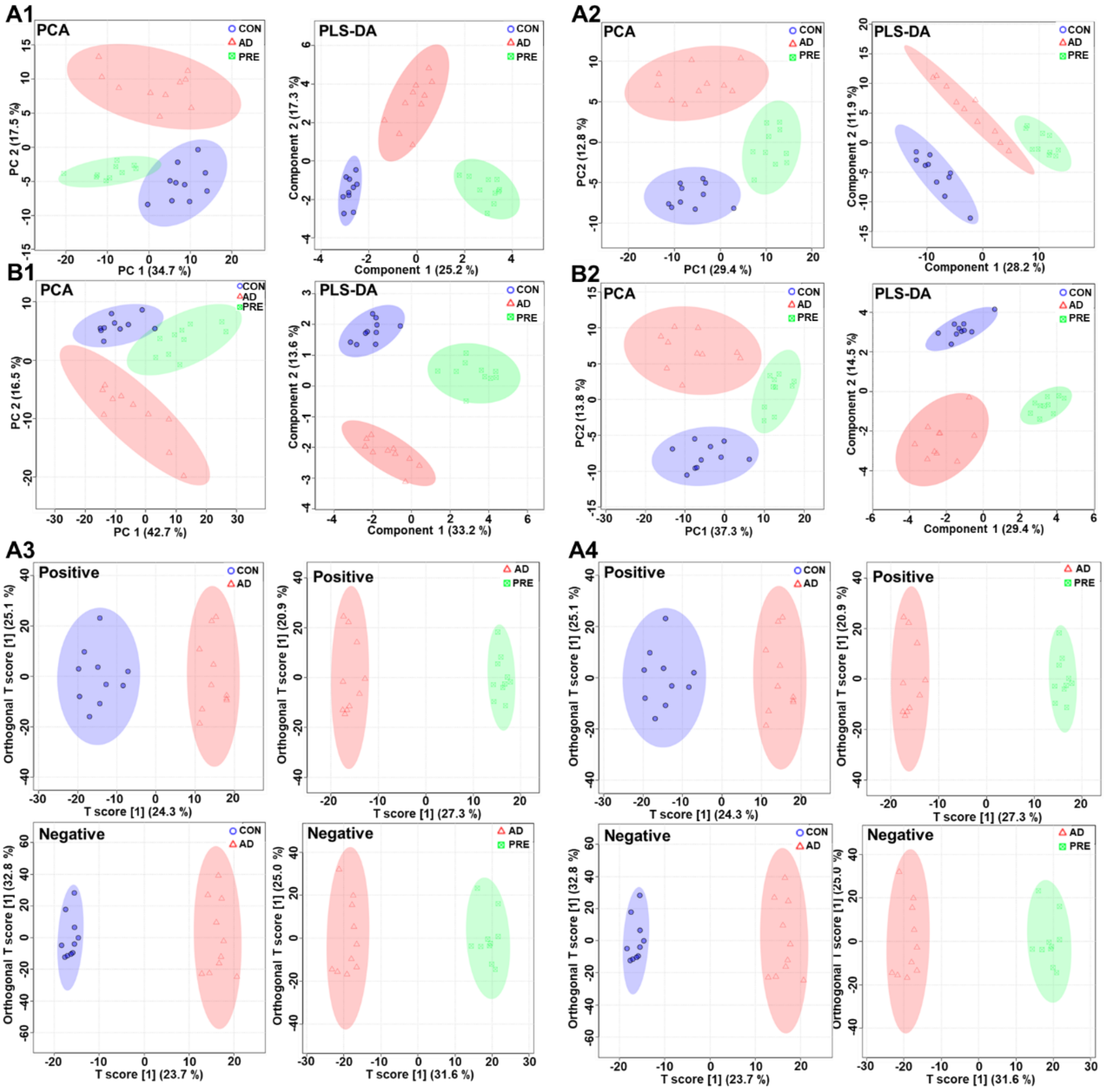

Figure S10. PCA and PLS-DA score plot of (**A1** and **B1**) urine and (**A2** and **B2**) faeces based on UPLC Q/TOF MS in (**A**) negative and (**B**) positive ion mode. OPLS-DA score plot of urine (**A3**) and faeces (**A4**) based on UPLC Q/TOF MS in negative and positive ion mode.

Figure S11

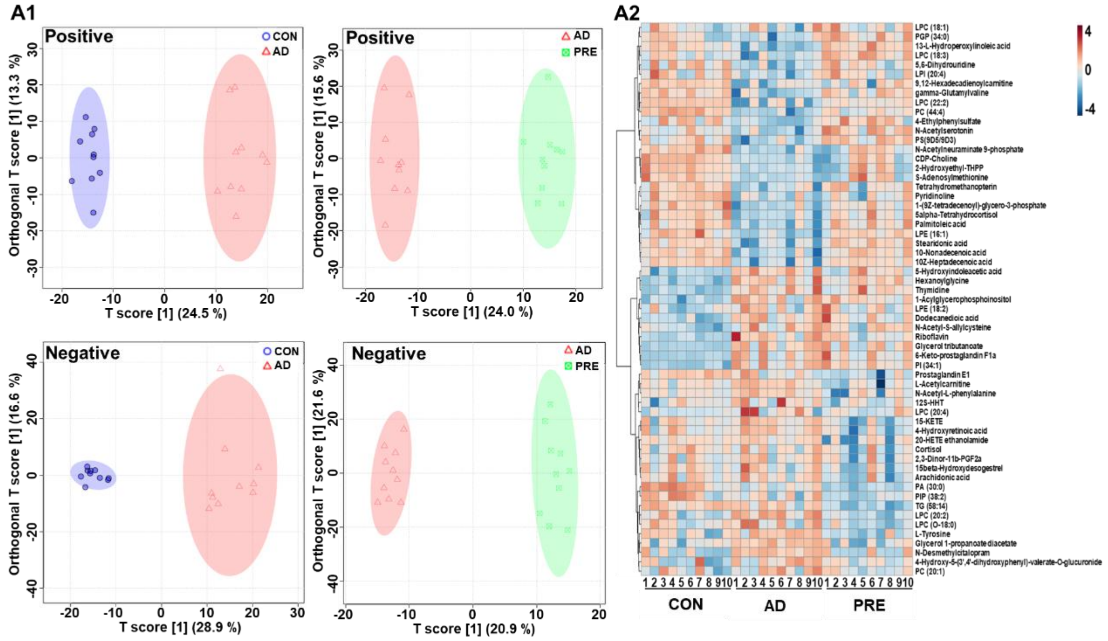

Figure S11. (**A1**) OPLS-DA score plot of blood based on UPLC Q/TOF MS in negative and positive ion mode. (**A2**) Heat map of differential metabolites by PRE reverses in the blood of AD mice.

Figure S12

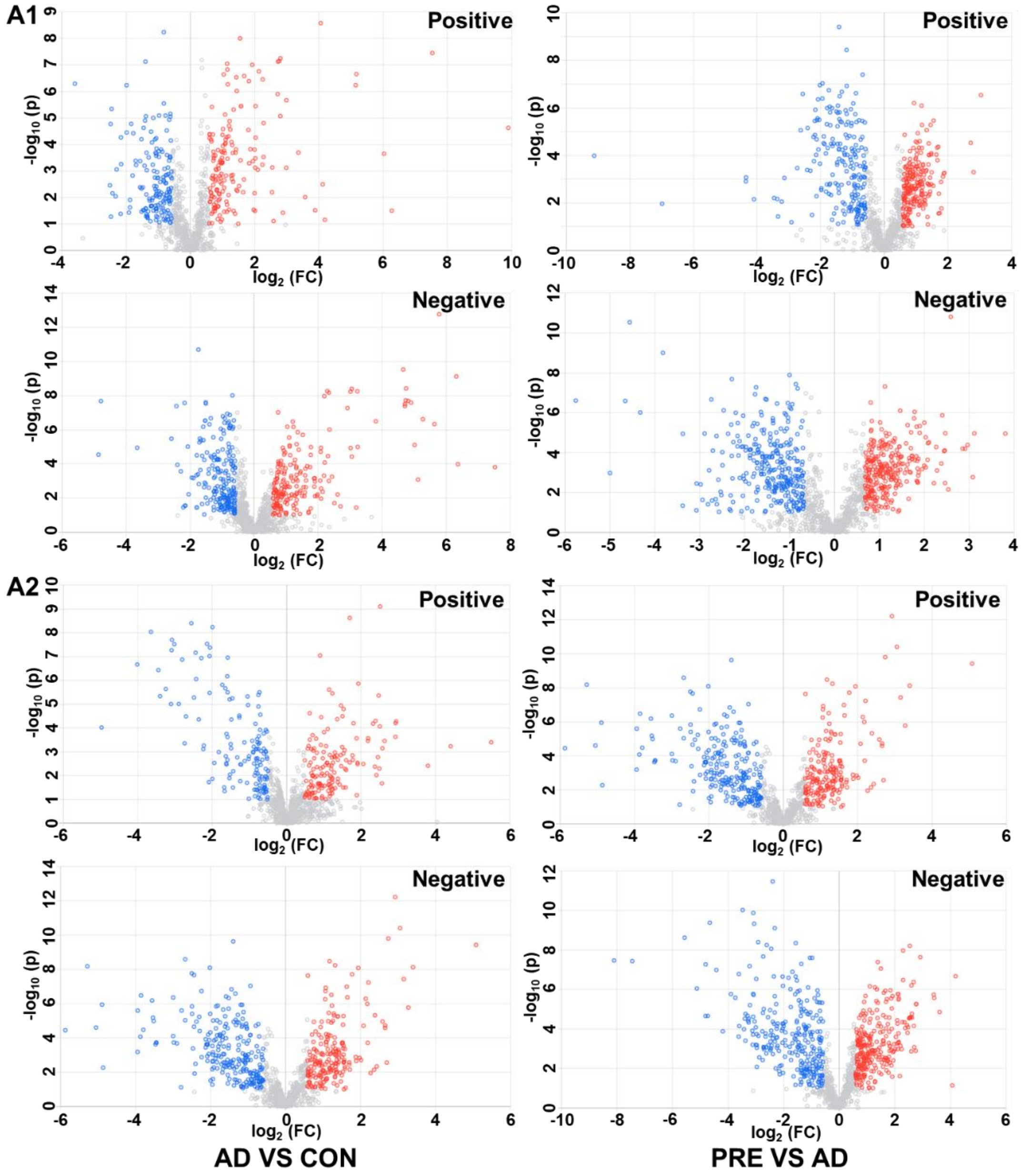

Figure S12. Volcano plot displayed differential metabolites with fold-change >1.5 in urine (**A1**) and faeces (**A2**) between AD and CON groups as well as PRE and AD groups.

Figure S13

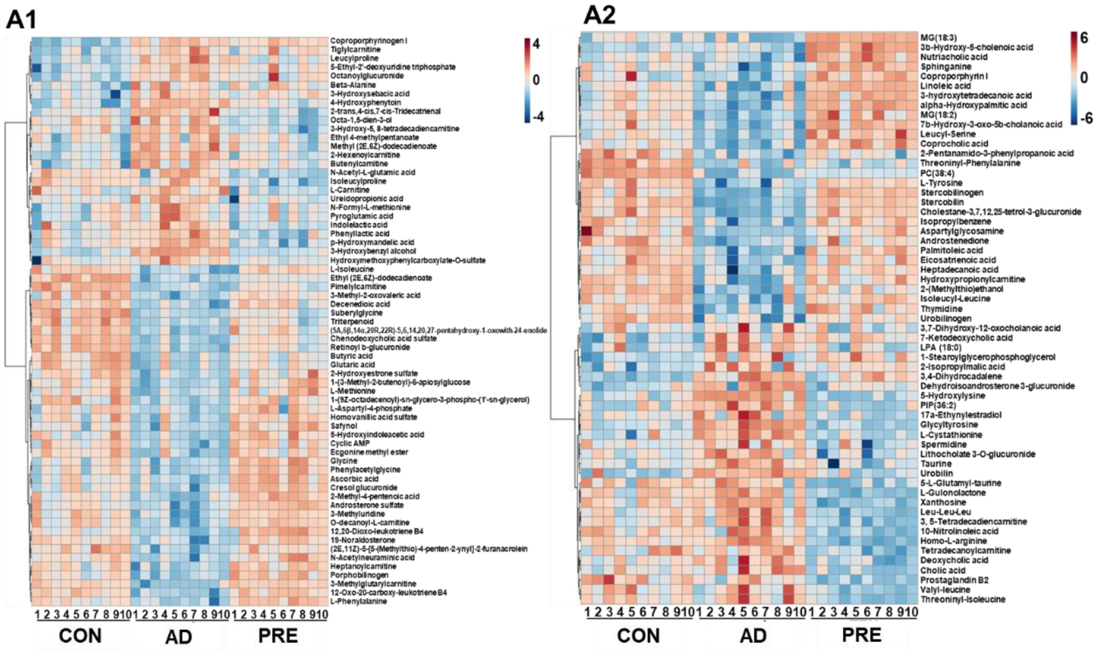

Figure S13. Heat map of differential metabolites by PRE reverses in urine (**A1**) and faeces (**A2**) of AD mice.

Figure S14

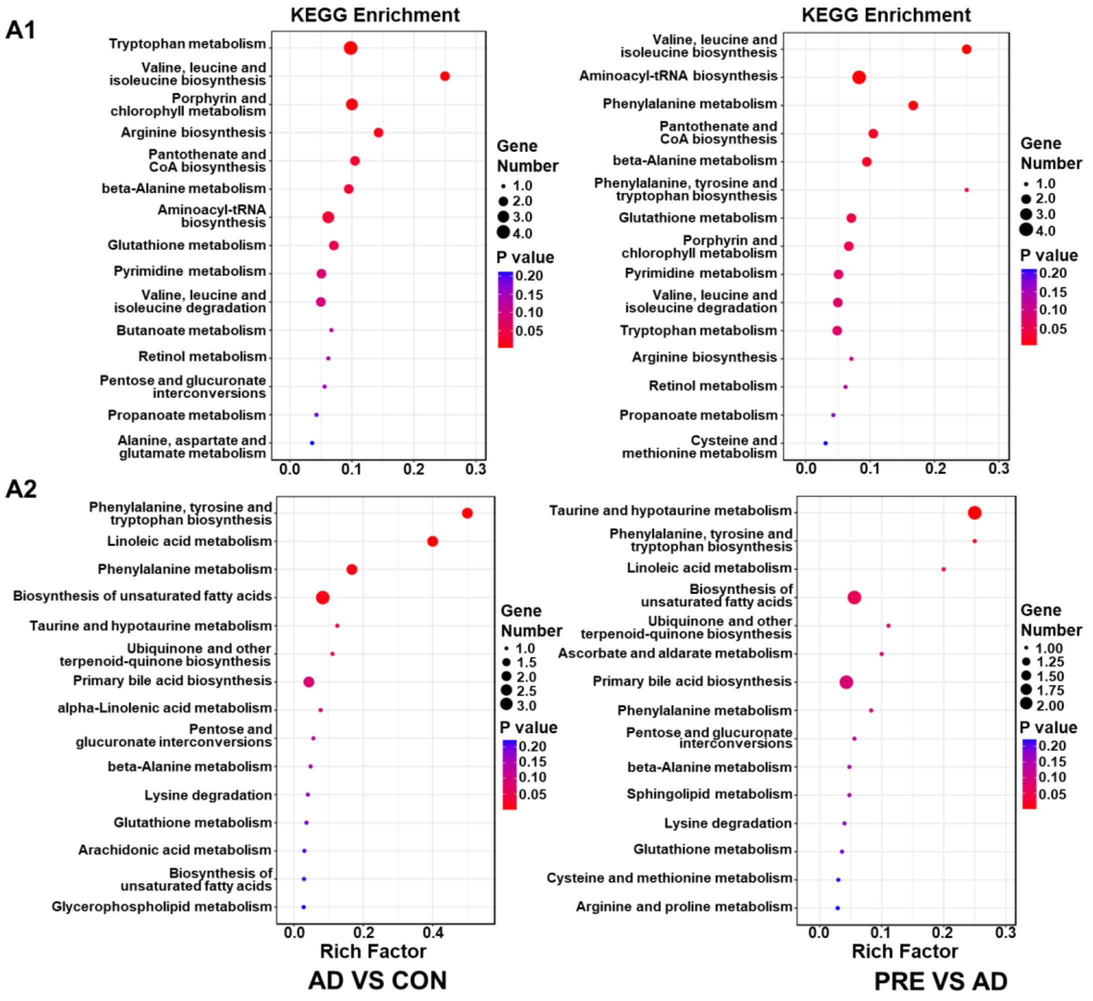

Figure S14. The enriched metabolic pathways for the differential metabolites in (**A1**) urine (**A1**) and faeces (**A2**) of AD model mice and PRE-treated mice.

Figure S15

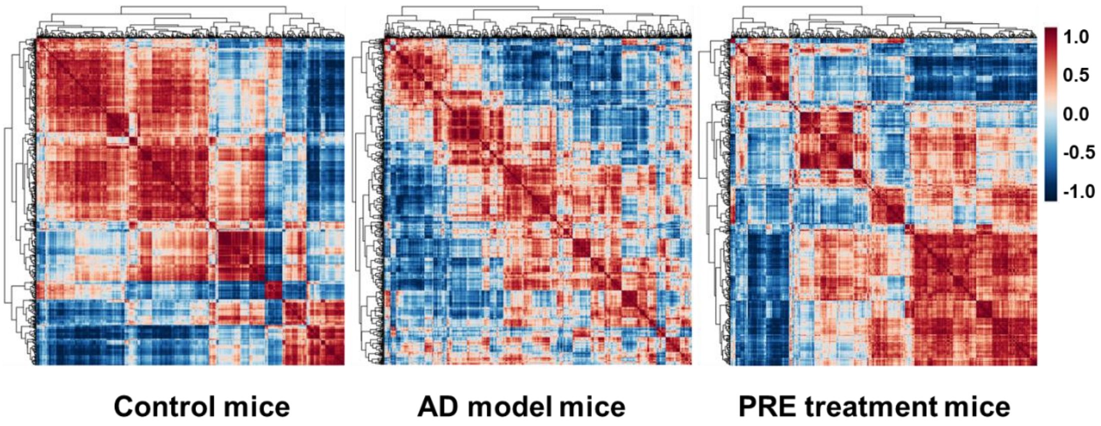

Figure S15. Correlation matrices of differential metabolites in 5 brain regions of three groups.

Figure S16

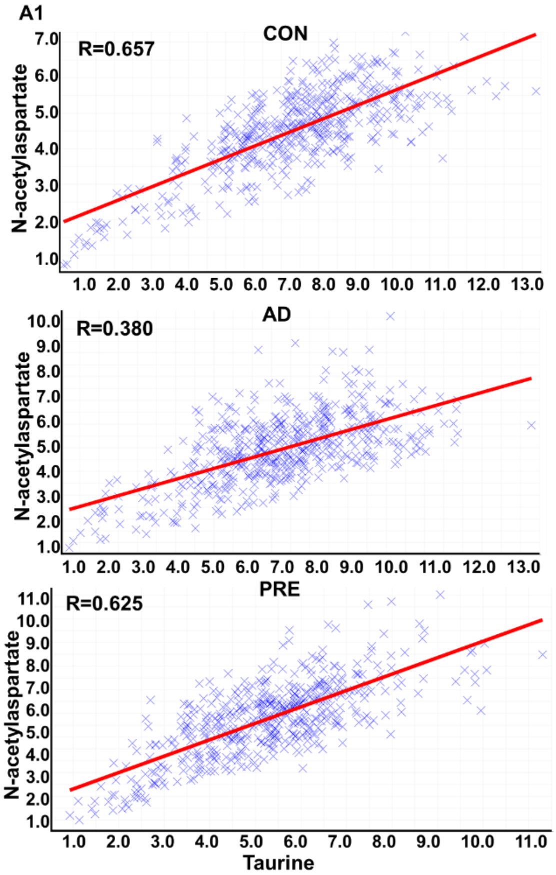

Figure S16. Correlation of taurine and N-acetylaspartate in Hp (**A1**) and whole brain (**A2**) of three groups of mice (n = 3 biological replicates in each group).

Figure S17

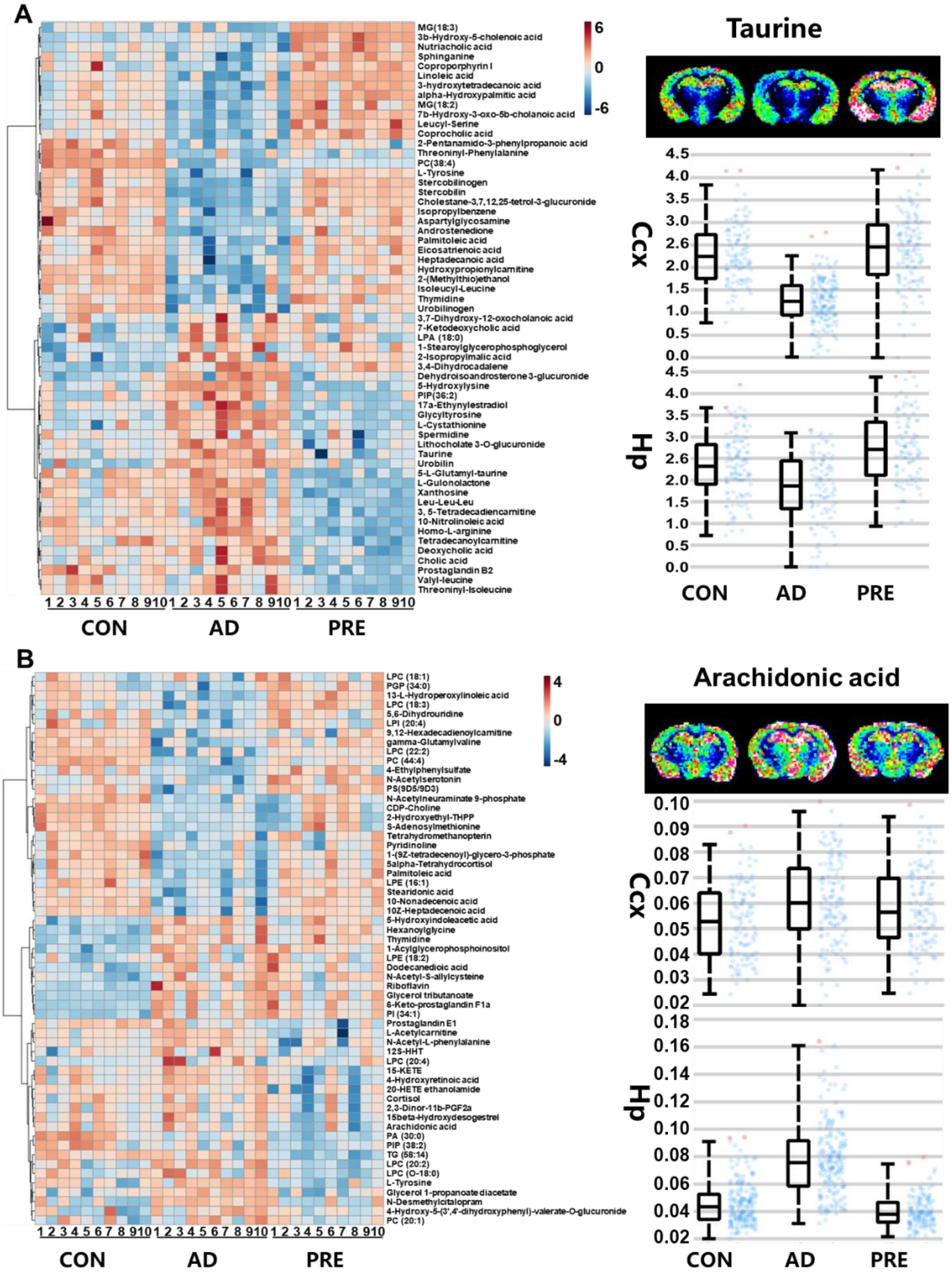

Figure S17. (**A**) The change trends of taurine in blood, Ccx and Hp regions of three groups. (**B**) The change trends of Arachidonic acid in faeces, Ccx and Hp regions of three group mice. Ccx, cerebral cortex; Hp, hippocampus.

Figure S18

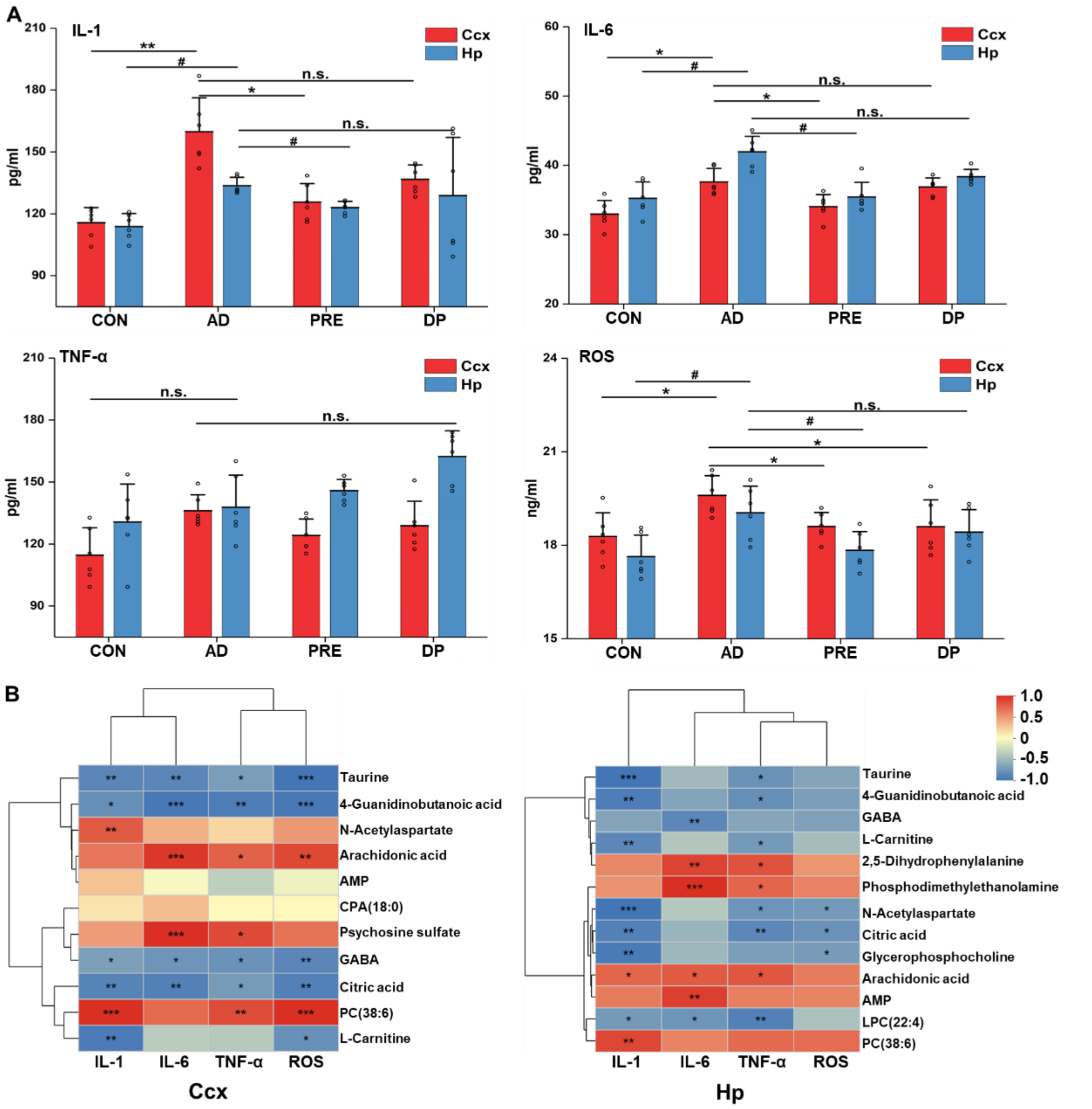

Figure S18. (**A**) PRE-treated improves the neuroinflammation in the AD mice brain. The levels of IL-1, IL-6, TNF-α, and ROS in the Ccx and Hp detected by ELISA. (**B**) The correlation matrices between key reverse metabolites and inflammatory factors in the Ccx and Hp regions. # p <0.05, * $p < 0.05$, ** $p < 0.01$, *** $p < 0.001$, and n.s.: no significant difference, by One-way ANOVA test. Ccx, cerebral cortex; Hp, hippocampus.

Table S1. Characterisation of chemical constituents in PRE

No.	tR (min)	Formula	Identification	Calculate (*m/z*)	Measured (*m/z*)	Diff. (ppm)	Fragment Ions (*m/z*)	Ion Form	Classification / Structure
	2.767	$C_{22}H_{30}O_{14}$	Sibiricose A5	517.1557	517.1566	-1.7403	341.109, 175.039, 160.842	[M−H]$^-$	Oligosaccharide esters / Steroid nucleus D
	3.516	$C_{19}H_{18}O_{10}$	Lancerin	405.0821	405.0824	-0.7406	243.028, 258.050, 285.039, 315.052	[M−H]$^-$	Xanthone/ Steroid nucleus E
#	3.566	$C_{24}H_{26}O_{14}$	Sibiricaxanthone A	537.1244	537.1258	-2.6065	405.082, 387.114 315.052, 267.031	[M−H]$^-$	Xanthone/ Steroid nucleus E
#	3.749	$C_{25}H_{28}O_{15}$	Polygalaxanthone III	567.1350	567.1357	-1.2343	447.115, 327.072, 315.050	[M−H]$^-$	Xanthone/ Steroid nucleus E
	3.832	$C_{20}H_{20}O_{11}$	Irisxanthone	435.0927	435.0929	-0.4597	345.062, 315.052, 272.032	[M−H]$^-$	Xanthone/ Steroid nucleus E
	3.849	$C_{20}H_{20}O_{11}$	7-O-Methylmangiferin	435.0927	435.0933	-1.3790	315.050, 272.030, 243.028	[M−H]$^-$	Xanthone/ Steroid nucleus E
#	3.882	$C_{24}H_{34}O_{15}$	Sibiricose A2	607.1874	607.1864	1.6469	561.180, 237.075	[M+COOH]$^-$	Oligosaccharide esters / Steroid nucleus D
	4.182	$C_{30}H_{36}O_{17}$	Tenuifoliside B	667.1874	667.1880	-0.8993	205.049, 137.023	[M−H]$^-$	Oligosaccharide esters / Steroid nucleus D
#	4.898	$C_{34}H_{42}O_{19}$	3,6'-disinapoyl sucrose	753.2242	753.2238	0.5311	547.165, 529.115, 205.049	[M−H]$^-$	Oligosaccharide esters / Steroid nucleus D
0	5.131	$C_{33}H_{40}O_{18}$	Arillanin A	723.2136	723.2142	-0.8296	547.166, 223.061, 205.051, 175.040, 160.016	[M−H]$^-$	Oligosaccharide esters / Steroid nucleus D
1	5.414	$C_{66}H_{84}O_{38}$	Tenuifoliose G	1483.4562	1483.4529	2.2245	753.223, 1039.312, 1337.383	[M−H]$^-$	Oligosaccharide esters / Steroid nucleus C

2	5.431	$C_{65}H_{82}O_{37}$	Tenuifoliose M	1453.4456	1453.4422	2.3393	1161.341, 1039.308	$[M-H]^-$	Oligosaccharide esters / Steroid nucleus C
3#	6.113	$C_{31}H_{38}O_{17}$	Tenuifoliside A	681.2031	681.2040	-1.3212	529.158, 443.119, 137.024	$[M-H]^-$	Oligosaccharide esters / Steroid nucleus D
4	6.513	$C_{55}H_{68}O_{31}$	Tenuifoliose S	1223.3666	1223.3663	0.2452	307.077, 631.189, 955.291, 1077.327	$[M-H]^-$	Oligosaccharide esters / Steroid nucleus C
5	6.729	$C_{56}H_{70}O_{32}$	Tenuifoliose T	1253.3772	1253.3774	-0.1596	1077.337, 955.298, 647.20, 307.086	$[M-H]^-$	Oligosaccharide esters / Steroid nucleus C
6	6.746	$C_{68}H_{86}O_{39}$	Tenuifoliose F	1525.4668	1525.4661	0.4589	1379.404, 1337.362, 1161.372	$[M-H]^-$	Oligosaccharide esters / Steroid nucleus C
7	6.829	$C_{67}H_{84}O_{38}$	Tenuifoliose L	1495.4562	1495.4522	2.6748	1349.386, 1203.367, 1161.376	$[M-H]^-$	Oligosaccharide esters / Steroid nucleus C
8	7.262	$C_{35}H_{44}O_{19}$	Tenuifoliside C	767.2398	767.2402	-0.5213	529.156, 367.102, 237.076, 223.061, 205.050	$[M-H]^-$	Oligosaccharide esters / Steroid nucleus D
9	7.378	$C_{58}H_{72}O_{33}$	Tenuifoliose C	1295.3878	1295.3892	-1.0808	1119.341, 997.310	$[M-H]^-$	Oligosaccharide esters / Steroid nucleus C
0	7.578	$C_{57}H_{72}O_{33}$	Tenuifoliose X	1283.3878	1283.3849	2.2596	1959.276, 193.048, 175.037	$[M-H]^-$	Oligosaccharide esters / Steroid nucleus C
1	7.595	$C_{59}H_{74}O_{34}$	Tenuifoliose P	1325.3983	1325.3980	0.2263	1265.579, 1119.345	$[M-H]^-$	Oligosaccharide esters / Steroid nucleus C
2	7.695	$C_{59}H_{72}O_{33}$	Tenuifoliose J	1307.3878	1307.3897	-1.4533	145.029, 163.039, 997.297, 1039.314, 1101.331, 1119.336, 1161.349, 1265.377	$[M-H]^-$	Oligosaccharide esters / Steroid nucleus C
3	7.795	$C_{57}H_{70}O_{32}$	Tenuifoliose K	1265.3772	1265.3754	1.4225	1173.446, 1119.338	$[M-H]^-$	Oligosaccharide esters / Steroid nucleus C
	8.444	$C_{53}H_{84}O_{24}$	Polygalasaponin	1103.5274	1103.5281	-0.6343	455.315, 425.862	$[M-H]^-$	Polygalasaponin /

4#			XXVIII						Steroid nucleus A
5	8.61	$C_{59}H_{72}O_{33}$	Tenuifoliose I	1307.3878	1307.3876	0.1530	1265.580, 1161.348	$[M-H]^-$	Oligosaccharide esters / Steroid nucleus C
6	8.827	$C_{60}H_{74}O_{34}$	Tenuifoliose B	1337.3983	1337.3986	-0.2243	1295.384, 1161.351, 1119.33, 1101.323, 997.305,145.029	$[M-H]^-$	Oligosaccharide esters / Steroid nucleus C
7	8.877	$C_{32}H_{40}O_{17}$	Methoxyl benzoyl-trimethoxy cinnamoyl sucrose	741.2242	741.2246	-0.5396	695.217, 543.168, 457.134, 237.076	$[M+COOH]^-$	Xanthone/ Steroid nucleus E
8	8.927	$C_{60}H_{74}O_{34}$	Tenuifoliose D	1337.3983	1337.3989	-0.4486	1039.313, 893.277, 997.306, 795.237, 1161.352, 175.037	$[M-H]^-$	Oligosaccharide esters / Steroid nucleus C
9	9.143	$C_{61}H_{76}O_{35}$	Tenuifoliose O	1367.4089	1367.4067	1.6089	1203.355, 175.036	$[M-H]^-$	Oligosaccharide esters / Steroid nucleus C
0#	9.193	$C_{61}H_{74}O_{34}$	Tenuifoliose H	1349.3973	1349.3984	-0.8152	1307.407, 1203.357, 145.028	$[M-H]^-$	Oligosaccharide esters / Steroid nucleus C
1	9.476	$C_{62}H_{76}O_{35}$	Tenuifoliose A	1379.4089	1379.4094	-0.3625	1203.359, 145.029	$[M-H]^-$	Oligosaccharide esters / Steroid nucleus C
2	9.742	$C_{63}H_{78}O_{36}$	Tenuifoliose N	1409.4194	1409.4186	0.5676	1191.373, 175.038	$[M-H]^-$	Oligosaccharide esters / Steroid nucleus C
3#	12.04	$C_{36}H_{56}O_{12}$	Tenuifolin	679.3693	679.3699	-0.8832	201.802, 409.232, 437.252, 501.280	$[M-H]^-$	Polygalasaponin / Steroid nucleus A
4	16.035	$C_{81}H_{122}O_{40}$	Onjisaponin S	1733.7434	1733.7428	0.3461	1703.779, 1509.694, 669.184, 425.309	$[M-H]^-$	Polygalasaponin / Steroid nucleus A
5	16.452	$C_{87}H_{130}O_{44}$	Onjisaponin Sg	1877.7856	1877.7864	-0.4260	1733.743, 1703.698, 1571.214, 509.680, 669.236, 425.307	$[M-H]^-$	Polygalasaponin / Steroid nucleus A
6	17.683	$C_{80}H_{120}O_{39}$	Senegin IV	1703.7328	1703.7324	0.2348	1479.674, 425.305	$[M-H]^-$	Polygalasaponin / Steroid nucleus A

7	18.216	$C_{86}H_{128}O_{43}$	Onjisaponin L	1847.7751	1847.7739	0.6494	425.307, 455.304	$[M-H]^-$	Polygalasaponin / Steroid nucleus A
8	18.582	$C_{77}H_{116}O_{37}$	Onjisaponin O	1631.7117	1631.7109	0.4903	425.302, 455.311	$[M-H]^-$	Polygalasaponin / Steroid nucleus A
9	19.132	$C_{87}H_{130}O_{45}$	Onjisaponin X	1893.7806	1893.7804	0.1056	1749.733, 669.215, 425.308	$[M-H]^-$	Polygalasaponin / Steroid nucleus A
0	19.149	$C_{87}H_{130}O_{45}$	Onjisaponin Ug	1893.7806	1893.7786	1.0561	1749.733, 1719.713, 669.226, 425.308	$[M-H]^-$	Polygalasaponin / Steroid nucleus A
1	19.681	$C_{76}H_{114}O_{37}$	Onjisaponin R	1617.6960	1617.6943	1.0509	455.311, 425.306	$[M-H]^-$	Polygalasaponin / Steroid nucleus A
2#	19.814	$C_{75}H_{112}O_{35}$	Onjisaponin B	1571.6906	1571.6911	-0.3181	455.317, 425.307	$[M-H]^-$	Polygalasaponin / Steroid nucleus A
3	20.497	$C_{70}H_{110}O_{37}$	Onjisaponin Pg	1541.6647	1541.6702	-3.5676	1397.539, 455.314, 425.304	$[M-H]^-$	Polygalasaponin / Steroid nucleus A
4	20.713	$C_{79}H_{118}O_{38}$	Polygalasaponin XXXII	1673.7223	1673.7206	1.0157	1449.659, 455.308, 425.305	$[M-H]^-$	Polygalasaponin / Steroid nucleus A
5	20.863	$C_{82}H_{122}O_{41}$	Onjisaponin V	1761.7383	1761.7387	-0.2270	425.304, 455.314, 1617.693	$[M-H]^-$	Polygalasaponin / Steroid nucleus A
6	21.146	$C_{80}H_{118}O_{38}$	Onjisaponin Ng	1685.7223	1685.7216	0.4153	455.309, 425.305	$[M-H]^-$	Polygalasaponin / Steroid nucleus A
7	21.196	$C_{76}H_{120}O_{40}$	Onjisaponin Qg	1671.7277	1671.7215	3.7088	1543.682, 1513.653	$[M-H]^-$	Polygalasaponin / Steroid nucleus A
8#	21.213	$C_{71}H_{106}O_{32}$	Onjisaponin Z	1469.6589	1469.6567	1.4969	1245.582, 1007.519, 455.317, 425.303	$[M-H]^-$	Polygalasaponin / Steroid nucleus A
9	21.479	$C_{85}H_{126}O_{42}$	Onjisaponin J	1817.7645	1817.7654	-0.4951	1593.718, 425.301	$[M-H]^-$	Polygalasaponin / Steroid nucleus A

0	21.662	$C_{83}H_{124}O_{42}$	Onjisaponin T	1791.7489	1791.7491	-0.1116	1731.711, 455.320, 425.307	$[M-H]^-$	Polygalasaponin / Steroid nucleus A
1	21.779	$C_{71}H_{106}O_{33}$	Onjisaponin E	1485.6538	1485.6543	-0.3366	425.303, 455.310	$[M-H]^-$	Polygalasaponin / Steroid nucleus A
2	22.428	$C_{90}H_{136}O_{45}$	Onjisaponin Tg	1935.8275	1935.8281	-0.3099	1791.745, 1761.746, 669.222, 425.306	$[M-H]^-$	Polygalasaponin / Steroid nucleus A
3#	22.628	$C_{75}H_{112}O_{36}$	Onjisaponin F	1587.6855	1587.6861	-0.3779	455.312, 425.305	$[M-H]^-$	Polygalasaponin / Steroid nucleus A
4	22.728	$C_{70}H_{104}O_{32}$	Onjisaponin G	1455.6432	1455.6423	0.6183	1231.589, 993.498, 455.319, 425.295	$[M-H]^-$	Polygalasaponin / Steroid nucleus A
5	22.811	$C_{70}H_{104}O_{32}$	Senegin II	1455.6432	1455.6438	-0.4122	455.313, 425.295	$[M-H]^-$	Polygalasaponin / Steroid nucleus A
6	22.894	$C_{69}H_{102}O_{30}$	Onjisaponin Y	1409.6377	1409.6365	0.8513	1187.601,1007.525, 455.317, 405.141	$[M-H]^-$	Polygalasaponin / Steroid nucleus A
7	24.01	$C_{81}H_{120}O_{40}$	Onjisaponin Fg	1731.7277	1731.7281	-0.2310	1587.684, 455.315, 425.303	$[M-H]^-$	Polygalasaponin / Steroid nucleus A
8	24.047	$C_{76}H_{112}O_{36}$	Onjisaponin Gg	1599.6855	1599.6788	4.1883	455.327, 425.305	$[M-H]^-$	Polygalasaponin / Steroid nucleus A
9	24.909	$C_{65}H_{96}O_{28}$	Onjisaponin TH	1323.6009	1323.5995	1.0577	1099.535, 455.320, 425.304	$[M-H]^-$	Polygalasaponin / Steroid nucleus A
0	27.789	$C_{63}H_{92}O_{26}$	Onjisaponin MF	1263.5798	1263.5778	1.5828	1039.533, 775.421, 455.318, 425.309	$[M-H]^-$	Polygalasaponin / Steroid nucleus A

# Compared with a reference standard.

Table S2. The regulated differential metabolites observed in Ccx region of PRE-treated AD mice

NO.	Measured m/z	Calculated m/z	Error (ppm)	Ion form	Compound	Molecular Formula	VIP	P value
1	124.007	124.0074	-3.226	$[M-H]^-$	Taurine	$C_2H_7NO_3S$	1.325319	0.001874
2	126.067	126.0667	2.380	$[M-H_2O-H]^-$	4-Guanidinobutanoic acid	$C_5H_{11}N_3O_2$	1.238811	0.003332
3	140.012	140.0114	4.285	$[M+K-2H]^-$	gamma-Aminobutyric acid	$C_4H_9NO_2$	1.241836	0.011443
4	162.113	162.1125	3.084	$[M+H]^+$	L-Carnitine	$C_7H_{15}NO_3$	1.526120	0.002583
5	166.088	166.0874	3.613	$[M-H]^-$	2,5-Dihydrophenylalanine	$C_9H_{13}NO_2$	1.272580	0.009476
6	168.044	168.0431	2.868	$[M-H]^-$	Phosphodimethylethanolamine	$C_4H_{12}NO_4P$	1.269277	0.009060
7	174.041	174.0403	4.022	$[M-H]^-$	N-Acetylaspartate	$C_6H_9NO_5$	1.285219	0.004027
8	191.020	191.0192	2.618	$[M-H]^-$	Citric acid	$C_6H_8O_7$	1.558795	0.004365
9	204.122	204.1230	-5.389	$[M+H]^+$	L-Acetylcarnitine	$C_9H_{17}NO_4$	1.438200	0.007786
10	255.232	255.2322	0.784	$[M-H]^-$	Palmitic acid	$C_{16}H_{32}O_2$	1.375555	0.009928
11	299.261	299.2586	8.020	$[M-H]^-$	Hydroxyoctadecanoic acid	$C_{18}H_{36}O_3$	1.301801	0.003340
12	303.233	303.2324	1.979	$[M-H]^-$	Arachidonic acid	$C_{20}H_{32}O_2$	1.375648	0.006059
13	306.075	306.0771	-7.841	$[M-H]^-$	Glutathione	$C_{10}H_{17}N_3O_6S$	1.274820	0.005661
14	346.056	346.0558	0.578	$[M-H]^-$	AMP	$C_{10}H_{14}N_5O_7P$	1.318221	0.000386
15	381.277	381.2775	-1.836	$[M+Na-2H]^-$	Tetracosatetraenoic acid (24:4)	$C_{24}H_{40}O_2$	1.148384	0.009857
16	391.224	391.2255	-4.343	$[M-H]^-$	CPA(16:0)	$C_{19}H_{37}O_6P$	1.293192	0.004920
17	419.260	419.2568	7.633	$[M-H]^-$	CPA(18:0)	$C_{21}H_{41}O_6P$	1.259823	0.007624
18	478.292	478.2939	-3.972	$[M-H]^-$	LPE(18:1)	$C_{23}H_{46}NO_7P$	1.108351	0.007389
19	500.279	500.2782	0.952	$[M-H]^-$	LPE(20:4)	$C_{25}H_{44}NO_7P$	1.194851	0.006015
20	522.288	522.2837	7.659	$[M-H]^-$	LPS(18:1)	$C_{24}H_{46}NO_9P$	1.277283	0.005301
21	540.283	540.2842	-2.221	$[M-H]^-$	Psychosine sulfate	$C_{24}H_{47}NO_{10}S$	1.127905	0.005991
22	647.466	647.4657	0.463	$[M-H]^-$	PA(32:0)	$C_{35}H_{69}O_8P$	1.249708	0.002080
23	695.461	695.4657	-6.758	$[M-H]^-$	PA(36:4)	$C_{39}H_{69}O_8P$	1.289004	0.003724

24	703.575	703.5749	0.213	$[M+H]^+$	SM(d36:1)	$C_{39}H_{79}N_2O_6P$	1.847549	0.011239
25	714.511	714.5079	4.339	$[M-H]^-$	PE(34:2)	$C_{39}H_{74}NO_8P$	1.327945	0.004177
26	716.525	716.5236	1.954	$[M-H]^-$	PE(34:1)	$C_{39}H_{76}NO_8P$	1.208231	0.08635
27	728.561	728.5594	2.196	$[M-H]^-$	PE(P-36:1)	$C_{41}H_{80}NO_7P$	1.099227	0.000218
28	746.512	746.5101	2.679	$[M-H]^-$	PE(P-38:6)	$C_{43}H_{74}NO_7P$	1.305372	0.021423
29	747.499	747.4965	3.077	$[M-H]^-$	PA(40:6)	$C_{43}H_{73}O_8P$	1.401294	0.005212
30	748.526	748.5286	-3.473	$[M-H]^-$	PE(P-38:5)	$C_{43}H_{76}NO_7P$	1.242518	0.010759
31	753.590	753.5881	2.521	$[M+Na]^+$	SM(d36:1)	$C_{41}H_{83}N_2O_6P$	1.749601	0.006202
32	760.515	760.5134	1.709	$[M-H]^-$	PS(34:1)	$C_{40}H_{76}NO_{10}P$	1.377893	0.005212
33	760.546	760.5492	-4.207	$[M-H]^-$	PS(O-35:1)	$C_{41}H_{80}NO_9P$	1.325203	0.002597
34	762.504	762.5079	-5.377	$[M-H]^-$	PE(38:6)	$C_{43}H_{74}NO_8P$	1.324161	0.006818
35	762.523	762.5291	-8.000	$[M-H]^-$	PS(34:0)	$C_{40}H_{78}NO_{10}P$	1.269040	0.005085
36	772.528	772.5286	-0.777	$[M-H]^-$	PE(P-40:7)	$C_{45}H_{76}NO_7P$	1.261872	0.011604
37	774.543	774.5437	-0.904	$[M-H_2O-H]^-$	PE(40:5)	$C_{45}H_{80}NO_8P$	1.164205	0.042837
38	786.534	786.5290	6.357	$[M-H]^-$	PS(36:2)	$C_{42}H_{78}NO_{10}P$	1.270727	0.005926
39	788.493	788.4991	-7.609	$[M+K]^+$	PE(P-38:5)	$C_{43}H_{76}NO_7P$	1.760772	0.015011
40	788.538	788.5447	-8.497	$[M-H]^-$	PS(36:1)	$C_{42}H_{80}NO_{10}P$	1.331470	0.012618
41	790.541	790.5392	1.897	$[M-H]^-$	PE(40:6)	$C_{45}H_{78}NO_8P$	1.329116	0.013152
42	804.525	804.5179	8.701	$[M-H_2O-H]^-$	PS(39:5)	$C_{45}H_{78}NO_{10}P$	1.402608	0.003905
43	822.543	822.5401	3.647	$[M-H]^-$	ST(t36:1)	$C_{42}H_{81}NO_{12}S$	1.354198	0.006101
44	832.516	832.5134	3.123	$[M-H]^-$	PS(40:7)	$C_{46}H_{76}NO_{10}P$	1.279219	0.006957
45	833.518	833.5185	-0.720	$[M-H]^-$	PI(34:2)	$C_{43}H_{79}O_{13}P$	1.321972	0.012781
46	834.530	834.5291	1.438	$[M-H]^-$	PS(40:6)	$C_{46}H_{78}NO_{10}P$	1.425295	0.001350
47	834.572	834.5765	-5.871	$[M-H]^-$	ST(d38:1)	$C_{44}H_{85}NO_{11}S$	1.317325	0.002903
48	837.557	837.5498	9.074	$[M-H]^-$	PI(34:0)	$C_{43}H_{82}O_{13}P$	1.424528	0.000922
49	844.532	844.5253	7.933	$[M+K]^+$	PC(38:6)	$C_{46}H_{80}NO_8P$	1.694266	0.005488

50	846.542	846.5401	2.717	$[M-H]^-$	ST(t38:3)	$C_{44}H_{81}NO_{12}S$	1.206683	0.016337
51	848.560	848.5558	4.832	$[M-H]^-$	ST(t38:1)	$C_{44}H_{85}NO_{12}S$	1.422119	0.001477
52	856.5128	856.5134	-0.705	$[M-H]^-$	PS(42:9)	$C_{48}H_{76}NO_{10}P$	1.707816	0.018960
53	857.520	857.5180	1.749	$[M-H]^-$	PI(36:4)	$C_{45}H_{79}O_{13}P$	1.396949	0.001498
54	860.594	860.5922	2.208	$[M-H]^-$	ST(40:2)	$C_{46}H_{87}NO_{11}S$	1.314450	0.004139
55	883.535	883.5342	0.566	$[M-H]^-$	PI(38:5)	$C_{47}H_{81}O_{13}P$	1.355988	0.010066
56	885.550	885.5493	0.903	$[M-H]^-$	PI(38:4)	$C_{47}H_{83}O_{13}P$	1.376164	0.007708
57	892.652	892.6553	-4.257	$[M-H]^-$	ST(d42:0)	$C_{48}H_{95}NO_{11}S$	1.340352	0.011582
58	900.570	900.5642	6.885	$[M+K-2H]^-$	ST(d40:1)	$C_{46}H_{89}NO_{11}S$	1.308292	0.011340
59	916.654	916.6548	-0.436	$[M-H]^-$	ST(44:2)	$C_{50}H_{95}NO_{11}S$	1.435081	0.000543

Table S3. The regulated differential metabolites observed in Hp region of PRE-treated AD mice

NO.	Measured m/z	Calculated m/z	Error (ppm)	Ion form	Compound	Molecular Formula	VIP	P value
1	124.007	124.0074	-3.226	$[M-H]^-$	Taurine	$C_2H_7NO_3S$	1.053782	0.008775
2	126.067	126.0667	2.380	$[M-H_2O-H]^-$	4-Guanidinobutanoic acid	$C_5H_{11}N_3O_2$	1.166831	0.000011
3	140.012	140.0114	4.285	$[M+K-2H]^-$	gamma-Aminobutyric acid	$C_4H_9NO_2$	1.187972	0.017799
4	162.113	162.1125	3.084	$[M+H]^+$	L-Carnitine	$C_7H_{15}NO_3$	1.219731	0.011484
5	166.088	166.0874	3.613	$[M-H]^-$	2,5-Dihydrophenylalanine	$C_9H_{13}NO_2$	1.265595	0.005001
6	168.044	168.0425	6.546	$[M-H]^-$	Phosphodimethylethanolamine	$C_4H_{12}NO_4P$	1.246547	0.007745
7	174.041	174.0403	4.022	$[M-H]^-$	N-Acetylaspartate	$C_6H_9NO_5$	1.306713	0.002394
8	191.020	191.0192	2.618	$[M-H]^-$	Citric acid	$C_6H_8O_7$	1.487556	0.004754
9	204.122	204.1230	-5.389	$[M+H]^+$	L-Acetylcarnitine	$C_9H_{17}NO_4$	1.319874	0.011941
10	222.091	222.0901	4.052	$[M+H-H_2O]^+$	Glycerophosphocholine	$C_8H_{20}NO_6P$	1.171987	0.002022
11	232.154	232.1543	-0.861	$[M+H]^+$	Butyrylcarnitine	$C_{11}H_{21}NO_4$	1.377736	0.009626
12	303.233	303.2324	1.979	$[M-H]^-$	Arachidonic acid	$C_{20}H_{32}O_2$	1.157625	0.001188
13	346.056	346.0558	0.578	$[M-H]^-$	AMP	$C_{10}H_{14}N_5O_7P$	1.281541	0.005415
14	524.279	524.2777	2.480	$[M-H]^-$	LPE(22:6)	$C_{27}H_{44}NO_7P$	1.273048	0.002270
15	540.283	540.2842	-2.221	$[M-H]^-$	Psychosine sulfate	$C_{24}H_{47}NO_{10}S$	1.136221	0.001527
16	552.307	552.3096	-4.708	$[M-H]^-$	LysoPE(24:6)	$C_{29}H_{48}NO_7P$	1.276574	0.003695
17	558.318	558.3196	-2.866	$[M-H_2O-H]^-$	PS(22:2)	$C_{28}H_{52}NO_9P$	1.308783	0.000664
18	610.322	610.3263	-7.045	$[M+K]^+$	LPC(22:4)	$C_{30}H_{54}NO_7P$	1.468595	0.000650
19	614.360	614.3576	3.744	$[M+K]^+$	LPC(22:2)	$C_{30}H_{58}NO_7P$	1.386943	0.008195
20	627.535	627.5353	-1.115	$[M+H-H_2O]^+$	DG(38:4)	$C_{41}H_{72}O_5$	1.397114	0.003645
21	647.466	647.4657	0.463	$[M-H]^-$	PA(32:0)	$C_{35}H_{69}O_8P$	1.137210	0.000926
22	703.575	703.5742	1.137	$[M+H]^+$	SM(d34:1)	$C_{39}H_{79}N_2O_6P$	1.000580	0.010188
23	715.581	715.5760	6.987	$[M-H]^-$	PE-Cer(38:1)	$C_{40}H_{81}N_2O_6P$	1.315434	0.001140

24	723.494	723.4970	-4.147	$[M-H]^-$	PA(38:4)	$C_{41}H_{73}O_8P$	1.237091	0.008224
25	725.506	725.5127	-9.235	$[M-H]^-$	PA(38:3)	$C_{41}H_{74}O_8P$	1.315245	0.000585
26	729.417	729.4114	7.677	$[M+K-2H]^-$	PG(30:1)	$C_{36}H_{69}O_{10}P$	1.263689	0.004034
27	732.555	732.5538	1.638	$[M+NH_4]^+$	PE(35:1)	$C_{40}H_{75}O_8P$	1.477878	0.001921
28	734.569	734.5694	-0.545	$[M+H]^+$	PC(32:0)	$C_{40}H_{80}NO_8P$	1.421127	0.003002
29	738.507	738.5073	-1.083	$[M-H]^-$	PE(36:4)	$C_{41}H_{74}NO_8P$	1.293996	0.000870
30	744.558	744.5542	5.104	$[M-H]^-$	PE(36:1)	$C_{41}H_{80}NO_8P$	1.250889	0.002031
31	746.512	746.5101	2.679	$[M-H]^-$	PE(P-38:6)	$C_{43}H_{74}NO_7P$	1.288670	0.001169
32	747.499	747.4965	3.077	$[M-H]^-$	PA(40:6)	$C_{43}H_{73}O_8P$	1.048955	0.028137
33	752.557	752.5593	-3.189	$[M-H]^-$	PE(P-38:3)	$C_{43}H_{80}NO_7P$	1.271243	0.003427
34	760.515	760.5134	1.709	$[M-H]^-$	PS(34:1)	$C_{40}H_{75}NO_{10}P$	1.366763	0.007718
35	762.523	762.5291	-8.000	$[M-H]^-$	PS(34:0)	$C_{40}H_{78}NO_{10}P$	1.111461	0.005009
36	766.538	766.5392	-1.435	$[M-H]^-$	PE(38:4)	$C_{43}H_{78}NO_8P$	1.292904	0.000703
37	768.559	768.5549	5.855	$[M-H]^-$	PE(38:3)	$C_{43}H_{80}NO_8P$	1.285979	0.001297
38	774.543	774.5437	-0.904	$[M-H_2O-H]^-$	PE(40:5)	$C_{45}H_{80}NO_8P$	1.242983	0.003662
39	776.559	776.5593	-0.644	$[M-H]^-$	PE(P-40:5)	$C_{45}H_{80}NO_7P$	1.239330	0.005639
40	790.541	790.5392	1.897	$[M-H]^-$	PE(40:6)	$C_{45}H_{78}NO_8P$	1.107544	0.008479
41	794.568	794.5699	-2.769	$[M-H]^-$	PE(40:4)	$C_{45}H_{82}NO_8P$	1.286870	0.001469
42	806.548	806.5452	2.852	$[M-H]^-$	3-O-Sulfogalactosylceramide (d36:1)	$C_{42}H_{81}NO_{11}S$	1.154461	0.024657
43	810.529	810.5290	-0.247	$[M-H]^-$	PS(38:4)	$C_{44}H_{77}NO_{10}P$	1.046533	0.017189
44	816.561	816.5549	7.715	$[M-H]^-$	PE(42:7)	$C_{47}H_{79}NO_8P$	1.180207	0.025694
45	818.579	818.5705	10.873	$[M-H]^-$	PE(42:6)	$C_{47}H_{81}NO_8P$	1.217514	0.012053
46	821.532	821.5338	-2.800	$[M-H]^-$	PG(18:0/22:6)	$C_{46}H_{79}O_{10}P$	1.125895	0.002475
47	822.543	822.5396	4.255	$[M-H]^-$	ST(t36:1)	$C_{42}H_{81}NO_{12}S$	1.153040	0.022614
48	832.516	832.5134	3.123	$[M-H]^-$	PS(40:7)	$C_{46}H_{76}NO_{10}P$	1.300688	0.000798
49	834.53	834.5291	1.438	$[M-H]^-$	PS(40:6)	$C_{46}H_{78}NO_{10}P$	1.016833	0.016326

50	836.606	836.6006	5.857	$[M+K]^+$	GlcCer(d16:2/24:0(2OH))	$C_{46}H_{87}NO_9$	1.062303	0.003699
51	844.606	844.6073	-1.302	$[M-H]^-$	PS(40:1)	$C_{46}H_{87}NO_{10}P$	1.079169	0.006916
52	850.574	850.5714	2.939	$[M-H]^-$	ST(t38:1)	$C_{44}H_{85}NO_{12}S$	1.172319	0.000110
53	856.5128	856.5134	-0.705	$[M-H]^-$	PS(42:9)	$C_{48}H_{76}NO_{10}P$	1.151945	0.000926
54	860.594	860.5922	2.208	$[M-H]^-$	ST(40:2)	$C_{46}H_{87}NO_{11}S$	1.037969	0.018144
55	862.609	862.6084	1.159	$[M-H]^-$	3-O-Sulfogalactosylceramide (d40:1)	$C_{46}H_{89}NO_{11}S$	1.156363	0.000585
56	864.628	864.6240	4.973	$[M-H]^-$	ST(40:0)	$C_{46}H_{91}NO_{11}S$	1.125527	0.003196
57	874.577	874.5714	6.174	$[M-H]^-$	ST(t40:3)	$C_{46}H_{85}NO_{12}S$	1.167802	0.000205
58	876.591	876.5871	4.563	$[M-H]^-$	ST(t40:2)	$C_{46}H_{87}NO_{12}S$	1.110030	0.004615
59	878.609	878.6027	7.284	$[M-H]^-$	ST(t40:1)	$C_{46}H_{89}NO_{12}S$	1.287279	0.003545
60	885.550	885.5493	0.903	$[M-H]^-$	PI(38:4)	$C_{47}H_{83}O_{13}P$	1.164004	0.017263
61	886.568	886.5603	8.911	$[M-H]^-$	PS(44:8)	$C_{50}H_{82}NO_{10}P$	1.138157	0.003298
62	888.626	888.6235	2.926	$[M-H]^-$	3-O-Sulfogalactosylceramide (d42:2)	$C_{48}H_{91}NO_{11}S$	1.258522	0.014260
63	892.652	892.6548	-3.697	$[M-H]^-$	ST(d42:0)	$C_{48}H_{95}NO_{11}S$	1.304789	0.002291
64	900.570	900.5642	6.885	$[M+K-2H]^-$	ST(d40:1)	$C_{46}H_{89}NO_{11}S$	1.141545	0.001798
65	904.618	904.6184	-0.630	$[M-H]^-$	ST(t42:2)	$C_{48}H_{91}NO_{12}S$	1.126605	0.003485
66	907.535	907.5342	0.551	$[M-H]^-$	PI(40:7)	$C_{49}H_{80}O_{13}P$	1.309616	0.000671
67	910.661	910.6662	-5.271	$[M+K]^+$	PC(42:1)	$C_{50}H_{98}NO_8P$	1.410221	0.005765
68	916.654	916.6548	-0.436	$[M-H]^-$	ST(44:2)	$C_{50}H_{95}NO_{11}S$	1.164575	0.000292
69	918.672	918.6704	1.742	$[M-H]^-$	ST(44:1)	$C_{50}H_{97}NO_{11}S$	1.167225	0.000179
70	920.692	920.6861	6.517	$[M-H]^-$	ST(44:0)	$C_{50}H_{98}NO_{11}S$	1.123666	0.003468
71	932.652	932.6511	0.536	$[M-H]^-$	ST(t44:2)	$C_{50}H_{95}NO_{12}S$	1.151433	0.000594
72	934.662	934.6659	-3.959	$[M-H]^-$	ST(t44:1)	$C_{50}H_{97}NO_{12}S$	1.125093	0.002640
73	1168.498	1168.4906	6.162	$[M+Na]^+$	CoA(26:0(3Ke))	$C_{47}H_{86}N_7O_{17}P_3S$	1.462145	0.000604
74	1749.827	1749.8226	2.286	$[M+2K-H]^+$	Ganglioside GD_2 (d36:2)	$C_{78}H_{136}N_4O_{34}$	1.424446	0.004484
75	1782.002	1782.0024	-0.404	$[M+Na]^+$	Ganglioside GD_2 (d42:1)	$C_{84}H_{150}N_4O_{34}$	1.385800	0.008030

Table S4. The regulated differential metabolites observed in Th region of PRE-treated AD mice

NO.	Measured m/z	Calculated m/z	Error (ppm)	Ion form	Compound	Molecular Formula	VIP	P value
1	162.113	162.1125	3.084	$[M+H]^+$	L-Carnitine	$C_7H_{15}NO_3$	1.099884	0.015908
2	166.088	166.0874	3.613	$[M-H]^-$	2,5-Dihydrophenylalanine	$C_9H_{13}NO_2$	1.293516	0.001331
3	170.034	170.0326	8.234	$[M+K]^+$	Creatine	$C_4H_9N_3O_2$	1.384347	0.001384
4	171.006	171.0064	-1.164	$[M-H]^-$	Glycerophosphoric acid	$C_3H_9O_6$	1.519043	0.005329
5	204.122	204.1230	-5.389	$[M+H]^+$	L-Acetylcarnitine	$C_9H_{17}NO_4$	1.151743	0.002881
6	222.091	222.0901	4.052	$[M+H-2H_2O]^+$	Glycerophosphocholine	$C_8H_{20}NO_6P$	1.195637	0.028643
7	240.121	240.1206	2.915	$[M+Na]^+$	Propionylcarnitine	$C_{10}H_{19}NO_4$	1.275137	0.000063
8	255.232	255.2322	0.784	$[M-H]^-$	Palmitic acid	$C_{16}H_{32}O_2$	1.308143	0.000182
9	264.191	264.1934	-8.327	$[M+Na]^+$	(4E,6E,d14:2)sphingosine	$C_{14}H_{27}NO_2$	1.012169	0.007107
10	281.249	281.2486	1.422	$[M-H]^-$	Oleic acid	$C_{18}H_{34}O_2$	1.259773	0.006738
11	296.068	296.0660	6.755	$[M+K]^+$	Glycerophosphocholine	$C_8H_{20}NO_6P$	1.300944	0.000952
12	303.233	303.2324	1.979	$[M-H]^-$	Arachidonic acid	$C_{20}H_{32}O_2$	1.066576	0.004744
13	346.056	346.0553	2.023	$[M-H]^-$	AMP	$C_{10}H_{14}N_5O_7P$	1.315042	0.000166
14	362.051	362.0501	2.486	$[M-H]^-$	GMP	$C_{10}H_{14}N_5O_8P$	1.483045	0.002564
15	540.283	540.2842	-2.221	$[M-H]^-$	Psychosine sulfate	$C_{24}H_{47}NO_{10}S$	1.164081	0.003350
16	552.307	552.3096	-4.708	$[M-H]^-$	LysoPE(24:6)	$C_{29}H_{48}NO_7P$	1.521990	0.009044
17	558.318	558.3196	-2.866	$[M-H_2O-H]^-$	PS(22:2)	$C_{28}H_{52}NO_9P$	1.311412	0.000123
18	582.296	582.2957	0.859	$[M+K]^+$	LPC(20:4)	$C_{28}H_{50}NO_9$	1.232111	0.004986
19	602.363	602.3582	7.969	$[M+K]^+$	LPC(22:6)	$C_{30}H_{50}NO_7P$	1.327727	0.000059
20	614.360	614.3576	3.744	$[M+K]^+$	LPC(22:2)	$C_{30}H_{58}NO_7P$	1.079799	0.009499
21	695.461	695.4657	-6.758	$[M-H]^-$	PA(36:4)	$C_{39}H_{69}O_8P$	1.541613	0.003917
22	714.511	714.5079	4.339	$[M-H]^-$	PE(34:2)	$C_{39}H_{73}NO_8P$	1.464886	0.007794

23	747.499	747.4965	3.077	$[M-H]^-$	PA(40:6)	$C_{43}H_{73}O_8P$	1.272300	0.002658
24	748.526	748.5286	-3.473	$[M-H]^-$	PE(P-38:5)	$C_{43}H_{75}NO_7P$	1.294554	0.000878
25	753.590	753.5881	2.521	$[M+Na]^+$	SM(d36:1)	$C_{41}H_{83}N_2O_6P$	1.132221	0.000577
26	756.496	756.4940	2.895	$[M+K]^+$	PE(34:1)	$C_{39}H_{76}NO_8P$	1.094364	0.000672
27	756.593	756.5913	2.247	$[M-H]^-$	PE(P-38:1)	$C_{43}H_{84}NO_7P$	1.574836	0.002237
28	766.538	766.5392	-1.435	$[M-H]^-$	PE(38:4)	$C_{43}H_{78}NO_8P$	1.161488	0.018307
29	792.492	792.4940	-2.915	$[M+K]^+$	PC(34:4)	$C_{42}H_{76}NO_8P$	1.259922	0.000880
30	795.532	795.5306	1.383	$[M+K-2H]^-$	PA(40:1)	$C_{43}H_{83}O_8P$	1.137549	0.006281
31	806.514	806.5097	4.947	$[M+K]^+$	PE(38:4)	$C_{43}H_{78}NO_8P$	1.379062	0.007314
32	806.548	806.5458	2.108	$[M-H]^-$	3-O-Sulfogalactosylceramide (d36:1)	$C_{42}H_{81}NO_{11}S$	1.280484	0.002201
33	816.561	816.5549	7.715	$[M-H]^-$	PE(42:7)	$C_{47}H_{79}NO_8P$	1.466274	0.017155
34	830.514	830.5097	4.828	$[M+K]^+$	PE(40:6)	$C_{45}H_{78}NO_8P$	1.351230	0.000215
35	833.518	833.5185	-0.720	$[M-H]^-$	PI(34:2)	$C_{43}H_{78}O_{13}P$	1.252568	0.003442
36	838.562	838.5603	2.504	$[M-H]^-$	PS(40:4)	$C_{46}H_{81}NO_{10}P$	1.370373	0.002990
37	846.542	846.5401	2.717	$[M-H]^-$	ST(t38:3)	$C_{44}H_{81}NO_{12}S$	1.560207	0.003537
38	848.560	848.5558	4.832	$[M-H]^-$	ST(t38:2)	$C_{44}H_{83}NO_{12}S$	1.608151	0.000491
39	860.594	860.5922	2.208	$[M-H]^-$	ST(40:2)	$C_{46}H_{87}NO_{11}S$	1.294204	0.000500
40	862.609	862.6084	1.159	$[M-H]^-$	ST(40:1)	$C_{46}H_{89}NO_{11}S$	1.285041	0.001940
41	864.628	864.6240	4.973	$[M-H]^-$	ST(40:0)	$C_{46}H_{91}NO_{11}S$	1.516079	0.008789
42	874.577	874.5714	6.174	$[M-H]^-$	ST(t40:3)	$C_{46}H_{85}NO_{12}S$	1.298440	0.000778
43	885.550	885.5498	0.339	$[M-H]^-$	PI(38:4)	$C_{47}H_{82}O_{13}P$	1.206089	0.010079
44	886.568	886.5603	8.911	$[M-H]^-$	PS(44:8)	$C_{50}H_{82}NO_{10}P$	1.297564	0.000813
45	888.626	888.6235	2.926	$[M-H]^-$	3-O-Sulfogalactosylceramide (d42:2)	$C_{48}H_{91}NO_{11}S$	1.482495	0.004522
46	892.652	892.6553	-4.257	$[M-H]^-$	ST(d42:0)	$C_{48}H_{95}NO_{11}S$	1.205306	0.007220
47	896.658	896.6505	8.364	$[M+K]^+$	PE(44:1)	$C_{49}H_{96}NO_8P$	1.007147	0.001980
48	900.570	900.5642	6.885	$[M+K-2H]^-$	ST(d40:1)	$C_{46}H_{89}NO_{11}S$	1.307548	0.000031

49	902.579	902.5793	-0.332	$[M+K-2H]^-$	ST(d40:0)	$C_{46}H_{91}NO_{11}S$	1.113061	0.005783
50	904.618	904.6184	-0.630	$[M-H]^-$	ST(t42:2)	$C_{48}H_{91}NO_{12}S$	1.389829	0.008300
51	916.654	916.6553	-0.982	$[M-H]^-$	ST(44:2)	$C_{50}H_{95}NO_{11}S$	1.281507	0.000648
52	918.672	918.6710	1.089	$[M-H]^-$	ST(44:1)	$C_{50}H_{97}NO_{11}S$	1.243058	0.005962
53	963.478	963.4761	2.076	$[M+K]^+$	PIP(P-36:3)	$C_{45}H_{82}O_{15}P_2$	1.240894	0.008347
54	982.550	982.5529	-3.226	$[M+H]^+$	CDP-DG(34:0)	$C_{46}H_{85}N_3O_{15}P_2$	1.175087	0.000161
55	984.549	984.5573	-8.125	$[M+NH_4]^+$	PIP(38:4)	$C_{47}H_{84}O_{16}P_2$	1.227496	0.000164
56	1544.872	1544.8694	1.812	$[M-H]^-$	Ganglioside GM_1 (36:1)	$C_{73}H_{131}N_3O_{31}$	1.294317	0.000251

Table S5. The regulated differential metabolites observed in Hth region of PRE-treated AD mice

NO.	Measured m/z	Calculated m/z	Error (ppm)	Ion form	Compound	Molecular Formula	VIP	P value
1	126.067	126.0667	2.380	$[M-H_2O-H]^-$	4-Guanidinobutanoic acid	$C_5H_{11}N_3O_2$	1.069105	0.021836
2	132.029	132.0302	-6.817	$[M-H]^-$	Aspartate	$C_4H_7NO_4$	1.199638	0.008738
3	140.012	140.0114	4.285	$[M+K-2H]^-$	gamma-Aminobutyric acid	$C_4H_9NO_2$	1.122287	0.018918
4	145.061	145.0618	-4.205	$[M-H]^-$	Glutamine	$C_5H_{10}N_2O_3$	1.145681	0.015219
5	162.113	162.1125	3.084	$[M+H]^+$	L-Carnitine	$C_7H_{15}NO_3$	1.238231	0.020838
6	174.041	174.0407	1.494	$[M-H]^-$	N-acetylaspatate	$C_6H_9NO_5$	1.191425	0.007308
7	191.020	191.0192	2.618	$[M-H]^-$	Citric acid	$C_6H_8O_7$	1.198340	0.000783
8	204.122	204.1230	-5.389	$[M+H]^+$	L-Acetylcarnitine	$C_9H_{17}NO_4$	1.431322	0.034052
9	222.091	222.0901	4.052	$[M+H-2H_2O]^+$	Glycerophosphocholine	$C_8H_{20}NO_6P$	1.330496	0.000484
10	232.156	232.1543	7.753	$[M+H]^+$	Butyrylcarnitine	$C_{11}H_{21}NO_4$	1.316667	0.001058
11	241.013	241.0119	3.826	$[M-H]^-$	Inositol cyclic phosphate	$C_6H_{11}O_8P$	1.187051	0.005011
12	259.021	259.0224	-5.571	$[M-H]^-$	Fructose 6-phosphate	$C_6H_{13}O_9P$	1.237298	0.003401
13	303.233	303.2324	1.979	$[M-H]^-$	Arachidonic acid	$C_{20}H_{32}O_2$	1.215821	0.026021
14	306.075	306.0771	-7.841	$[M-H]^-$	Glutathione	$C_{10}H_{17}N_3O_6S$	1.216053	0.002910
15	346.056	346.0558	0.578	$[M-H]^-$	AMP	$C_{10}H_{14}N_5O_7P$	1.228977	0.003313
16	462.299	462.2984	0.433	$[M-H_2O-H]^-$	LPE(18:0)	$C_{23}H_{48}NO_7P$	1.199155	0.005862
17	478.292	478.2939	-3.972	$[M-H]^-$	LPE(18:1)	$C_{23}H_{46}NO_7P$	1.245016	0.001539
18	602.363	602.3582	7.969	$[M+K]^+$	LPC(22:6)	$C_{30}H_{50}NO_7P$	1.332538	0.000119
19	610.322	610.3275	-9.012	$[M+K]^+$	LPC(22:4)	$C_{30}H_{54}NO_7P$	1.694150	0.000567
20	618.357	618.3889	-51.101	$[M+K]^+$	LPC(22:0)	$C_{30}H_{62}NO_7P$	1.321858	0.002734
21	673.479	673.4814	-3.564	$[M-H]^-$	PA(34:1)	$C_{37}H_{71}O_8P$	1.238703	0.002089
22	703.575	703.5742	1.137	$[M+H]^+$	SM(d34:1)	$C_{39}H_{79}N_2O_6P$	1.413615	0.040586
23	716.525	716.5236	1.954	$[M-H]^-$	PE(34:1)	$C_{39}H_{76}NO_8P$	1.186560	0.008481

24	731.605	731.6061	-1.504	$[M+H]^+$	SM(d36:1)	$C_{41}H_{83}N_2O_6P$	1.339825	0.000108
25	732.555	732.5538	1.638	$[M+NH_4]^+$	PE(35:1)	$C_{40}H_{75}O_8P$	1.682330	0.001125
26	744.558	744.5542	5.104	$[M-H]^-$	PE(36:1)	$C_{41}H_{80}NO_8P$	1.206503	0.004560
27	747.499	747.4965	3.077	$[M-H]^-$	PA(40:6)	$C_{43}H_{72}O_8P$	1.122805	0.008539
28	750.547	750.5437	4.397	$[M-H]^-$	PE(P-38:4)	$C_{43}H_{78}NO_7P$	1.185230	0.007494
29	756.556	756.5538	2.908	$[M+H]^+$	PC(34:3)	$C_{42}H_{78}NO_8P$	1.314201	0.001530
30	762.601	762.6007	0.656	$[M+H]^+$	PC(34:0)	$C_{42}H_{84}NO_8P$	1.245160	0.009109
31	762.523	762.5291	-8.000	$[M-H]^-$	PS(34:0)	$C_{40}H_{78}NO_{10}P$	1.208273	0.002517
32	764.529	764.5236	6.409	$[M-H]^-$	PE(38:5)	$C_{43}H_{76}NO_8P$	1.220534	0.003827
33	766.538	766.5392	-1.435	$[M-H]^-$	PE(38:4)	$C_{43}H_{78}NO_8P$	1.263872	0.008431
34	770.577	770.5705	7.786	$[M-H]^-$	PE(38:2)	$C_{43}H_{82}NO_8P$	1.220849	0.004530
35	788.538	788.5447	-8.497	$[M-H]^-$	PS(36:1)	$C_{42}H_{79}NO_{10}P$	1.263872	0.004265
36	790.541	790.5392	1.897	$[M-H]^-$	PE(40:6)	$C_{45}H_{78}NO_8P$	1.298234	0.022293
37	792.492	792.4940	-2.915	$[M+K]^+$	PC(34:4)	$C_{42}H_{76}NO_8P$	1.667083	0.001066
38	794.547	794.5461	1.259	$[M+K]^+$	PC(34:3)	$C_{42}H_{78}NO_8P$	1.334855	0.058661
39	794.568	794.5699	-2.769	$[M-H]^-$	PE(40:4)	$C_{45}H_{82}NO_8P$	1.185954	0.007940
40	806.548	806.5458	2.108	$[M-H]^-$	3-O-Sulfogalactosylceramide (d36:1)	$C_{42}H_{81}NO_{11}S$	1.215686	0.000360
41	810.529	810.5290	-0.247	$[M-H]^-$	PS(38:4)	$C_{44}H_{77}NO_{10}P$	1.215686	0.032364
42	838.613	838.6161	-3.697	$[M+H]^+$	PI-Cer(d38:0)	$C_{44}H_{88}NO_{11}P$	1.617635	0.004582
43	844.532	844.5253	7.933	$[M+K]^+$	PC(38:6)	$C_{46}H_{80}NO_8P$	1.491737	0.018759
44	848.560	848.5558	4.832	$[M-H]^-$	ST(t38:1)	$C_{45}H_{92}NO_7P$	1.194454	0.000140
45	850.574	850.5714	2.939	$[M-H]^-$	ST(38:1(2OH))	$C_{44}H_{85}NO_{12}S$	1.194454	0.008723
46	856.5128	856.5134	-0.705	$[M-H]^-$	PS(42:9)	$C_{48}H_{76}NO_{10}P$	1.363913	0.003485
47	860.594	860.5922	2.208	$[M-H]^-$	ST(40:2)	$C_{46}H_{87}NO_{11}S$	1.225511	0.002043
48	862.609	862.6084	1.159	$[M-H]^-$	3-O-Sulfogalactosylceramide (40:1)	$C_{46}H_{89}NO_{11}S$	1.354615	0.007417
49	863.560	863.5655	-6.369	$[M-H]^-$	PI(36:1)	$C_{45}H_{85}O_{13}P$	1.199819	0.005428

50	874.577	874.5714	6.174	$[M-H]^-$	ST(t40:3)	$C_{46}H_{85}NO_{12}S$	1.275469	0.016424
51	885.550	885.5493	0.903	$[M-H]^-$	PI(38:4)	$C_{47}H_{83}O_{13}P$	1.209584	0.003399
52	886.568	886.5603	8.911	$[M-H]^-$	PS(44:8)	$C_{50}H_{82}NO_{10}P$	1.304499	0.001998
53	892.652	892.6548	-3.697	$[M-H]^-$	ST(d42:0)	$C_{48}H_{95}NO_{11}S$	1.196636	0.004812
54	904.618	904.6184	-0.630	$[M-H]^-$	ST(t42:2)	$C_{48}H_{91}NO_{12}S$	1.187339	0.006870
55	916.654	916.6553	-0.982	$[M-H]^-$	ST(44:2)	$C_{50}H_{95}NO_{11}S$	1.215410	0.002030
56	918.672	918.6710	1.089	$[M-H]^-$	ST(44:1)	$C_{50}H_{97}NO_{11}S$	1.336780	0.005149
57	1243.795	1243.8168	-17.737	$[M+K]^+$	Ganglioside GA_2 (d44:1)	$C_{64}H_{120}N_2O_{18}$	1.296350	0.004771
58	1297.732	1297.7367	-3.545	$[M+2Na-H]^+$	Ganglioside GA_1 (d36:2)	$C_{62}H_{112}N_2O_{23}$	1.327941	0.000775
59	1544.872	1544.8694	1.812	$[M-H]^-$	Ganglioside GM_1 (36:1)	$C_{73}H_{131}N_3O_{31}$	1.144346	0.000969

Table S6. The regulated differential metabolites observed in Cc region of PRE-treated AD mice

NO.	Measured m/z	Calculated m/z	Error (ppm)	Ion form	Compound	Molecular Formula	VIP	P value
1	87.008	87.0088	-6.896	$[M-H]^-$	pyruvate	$C_3H_4O_3$	1.329004	0.011051
2	104.107	104.1075	-5.763	$[M+H]^+$	Choline	$C_5H_{14}NO$	1.559185	0.000893
3	145.061	145.0612	0.000	$[M-H]^-$	Glutamine	$C_5H_{10}N_2O_3$	1.223492	0.005530
4	166.088	166.0874	3.613	$[M-H]^-$	2,5-Dihydrophenylalanine	$C_9H_{13}NO_2$	1.157110	0.021573
5	168.044	168.0425	6.546	$[M-H]^-$	Phosphodimethylethanolamine	$C_4H_{12}NO_4P$	1.154789	0.017343
6	174.041	174.0403	4.022	$[M-H]^-$	N-Acetylaspartate	$C_6H_9NO_5$	1.171816	0.008821
7	204.122	204.1230	-5.389	$[M+H]^+$	L-Acetylcarnitine	$C_9H_{17}NO_4$	1.640276	0.033559
8	214.048	214.0491	-5.139	$[M-H]^-$	Glycerylphosphorylethanolamine	$C_5H_{14}NO_6P$	1.228516	0.002535
9	255.232	255.2322	0.784	$[M-H]^-$	Palmitic acid	$C_{16}H_{32}O_2$	1.346961	0.008091
10	296.068	296.0660	6.755	$[M+K]^+$	Glycerophosphocholine	$C_8H_{20}NO_6P$	1.610355	0.026579
11	306.075	306.0771	-7.841	$[M-H]^-$	Glutathione	$C_{10}H_{17}N_3O_6S$	1.244747	0.000979
12	346.056	346.0553	2.023	$[M-H]^-$	AMP	$C_{10}H_{14}N_5O_7P$	1.239687	0.002209
13	403.264	403.2616	5.852	$[M-H_2O-H]^-$	PA(P-18:0)	$C_{21}H_{43}O_6P$	1.188534	0.011659
14	462.296	462.2984	-5.191	$[M-H_2O-H]^-$	PE(18:0)	$C_{23}H_{48}NO_7P$	1.129898	0.030062
15	564.247	564.2487	-3.013	$[M+K]^+$	LPE(22:6)	$C_{27}H_{44}NO_7P$	1.564106	0.008072
16	714.511	714.5079	4.339	$[M-H]^-$	PE(34:2)	$C_{39}H_{73}NO_8P$	1.444024	0.000188
17	715.581	715.5760	6.987	$[M-H]^-$	PE-Cer(38:1)	$C_{40}H_{81}N_2O_6P$	1.169682	0.010752
18	724.532	724.5286	4.693	$[M-H]^-$	PE(O-36:4)	$C_{41}H_{75}NO_7P$	1.249188	0.001025
19	739.517	739.5151	2.569	$[M+K]^+$	SM(d34:2)	$C_{39}H_{77}N_2O_6P$	1.522836	0.009618
20	744.558	744.5542	5.104	$[M-H]^-$	PE(36:1)	$C_{41}H_{80}NO_8P$	1.178261	0.017460
21	746.512	746.5101	2.679	$[M-H]^-$	PE(P-38:6)	$C_{43}H_{74}NO_7P$	1.173337	0.016115
22	750.443	750.4471	-5.677	$[M+K]^+$	PE(34:4)	$C_{39}H_{70}NO_8P$	1.579319	0.002094

23	756.496	756.4940	2.631	[M+K]$^{+}$	PE(34:1)	$C_{39}H_{76}NO_8P$	1.237328	0.002416
24	760.495	760.4923	3.156	[M-H]$^{-}$	PE(38:7)	$C_{43}H_{71}NO_8P$	1.298603	0.018003
25	762.523	762.5291	-8.000	[M-H]$^{-}$	PS(34:0)	$C_{40}H_{78}NO_{10}P$	1.364084	0.006723
26	764.529	764.5236	6.409	[M-H]$^{-}$	PE(38:5	$C_{43}H_{76}NO_8P$	1.274826	0.020698
27	788.538	788.5447	-8.497	[M-H]$^{-}$	PS(36:1)	$C_{42}H_{79}NO_{10}P$	1.424445	0.000935
28	802.510	802.5023	9.844	[M-H$_2$O-H]$^{-}$	PS(39:6)	$C_{45}H_{76}NO_{10}P$	1.337172	0.010550
29	806.548	806.5452	2.852	[M-H]$^{-}$	3-O-Sulfogalactosylceramide (d36:1)	$C_{42}H_{81}NO_{11}S$	1.161632	0.020406
30	810.529	810.5290	-0.247	[M-H]$^{-}$	PS(38:4)	$C_{44}H_{77}NO_{10}P$	1.092836	0.013651
31	821.532	821.5338	-2.800	[M-H]$^{-}$	PG(18:0/22:6)	$C_{46}H_{79}O_{10}P$	1.322184	0.014626
32	822.543	822.5396	4.255	[M-H]$^{-}$	ST(t36:1)	$C_{42}H_{81}NO_{12}S$	1.444091	0.000442
33	832.516	832.5134	3.123	[M-H]$^{-}$	PS(40:7)	$C_{46}H_{75}NO_{10}P$	1.290958	0.023347
34	833.518	833.5185	-0.720	[M-H]$^{-}$	PI(34:2)	$C_{43}H_{78}O_{13}P$	1.320221	0.017014
35	837.557	837.5498	9.074	[M-H]$^{-}$	PI(34:0)	$C_{43}H_{82}O_{13}P$	1.274532	0.023033
36	838.562	838.5603	2.504	[M-H]$^{-}$	PS(40:4)	$C_{46}H_{81}NO_{10}P$	1.180205	0.018998
37	844.551	844.5492	2.250	[M-H$_2$O-H]$^{-}$	PS(42:6)	$C_{48}H_{82}NO_{10}P$	1.215403	0.007546
38	846.542	846.5401	2.717	[M-H]$^{-}$	ST(t38:3)	$C_{44}H_{81}NO_{12}S$	1.362976	0.008040
39	856.588	856.5851	3.38	[M-H]$^{+}$	PC(42:9)	$C_{50}H_{82}NO_8P$	1.198816	0.015726
40	857.52	857.518	1.749	[M-H]$^{-}$	PI(36:4)	$C_{45}H_{79}O_{13}P$	1.394929	0.002811
41	860.594	860.5922	2.208	[M-H]$^{-}$	ST(40:2)	$C_{46}H_{87}NO_{11}S$	1.187238	0.010789
42	862.609	862.6084	1.159	[M-H]$^{-}$	3-O-Sulfogalactosylceramide (40:1)	$C_{46}H_{89}NO_{11}S$	1.193713	0.011954
43	876.591	876.5871	4.563	[M-H]$^{-}$	ST(t40:2)	$C_{46}H_{87}NO_{12}S$	1.189927	0.018044
44	878.609	878.6027	7.284	[M-H]$^{-}$	ST(t40:1)	$C_{46}H_{89}NO_{12}S$	1.259233	0.002195
45	888.626	888.6235	2.926	[M-H]$^{-}$	3-O-Sulfogalactosylceramide (d42:2)	$C_{48}H_{91}NO_{11}S$	1.449294	0.000138
46	890.636	890.6391	-3.593	[M-H]$^{-}$	3-O-Sulfogalactosylceramide (d42:1)	$C_{48}H_{93}NO_{11}S$	1.430094	0.000720
47	892.652	892.6548	-3.697	[M-H]$^{-}$	ST(d42:0)	$C_{48}H_{95}NO_{11}S$	1.157943	0.020758
48	900.570	900.5642	6.885	[M+K-2H]$^{-}$	3-O-Sulfogalactosylceramide (d40:1)	$C_{46}H_{89}NO_{11}S$	1.355631	0.009462

49	902.579	902.5793	-0.332	$[M+K-2H]^-$	ST(d40:0)	$C_{46}H_{91}NO_{11}S$	1.335032	0.011106
50	904.618	904.6184	-0.630	$[M-H]^-$	ST(t42:2)	$C_{48}H_{91}NO_{12}S$	1.236339	0.002016
51	907.535	907.5342	0.551	$[M-H]^-$	PI(40:7)	$C_{49}H_{80}O_{13}P$	1.255611	0.003828
52	920.711	920.7103	0.652	$[M+H]^+$	PC(46:5)	$C_{54}H_{98}NO_8P$	1.701692	0.004322
53	934.662	934.6659	-3.959	$[M-H]^-$	ST(t44:1)	$C_{50}H_{97}NO_{12}S$	1.354791	0.008544

Table S7. The regulated differential metabolites observed in blood of PRE-treated AD mice

NO.	Retention time (min)	Formula	Measured m/z	Theoretical m/z	Delta (ppm)	Metabolite	p value	VIP	Trend	ESI Model
1	0.762	$C_9H_{11}NO_3$	180.0664	180.0661	1.6661	L-Tyrosine	5.43E-05	1.567663	Down	-
2	1.504	$C_{10}H_9NO_3$	190.0508	190.0504	2.1047	5-Hydroxyindoleacetic acid	6.02E-05	1.722563	Down	-
3	2.445	$C_8H_{10}O_4S$	201.0225	201.0222	1.4924	4-Ethylphenylsulfate	0.00080071	1.523725	Up	-
4	2.068	$C_9H_{17}NO_4$	202.1083	202.1079	1.9791	L-Acetylcarnitine	8.34E-05	1.699133	Down	-
5	1.629	$C_{12}H_{14}N_2O_2$	219.1139	219.1134	2.2819	N-Acetylserotonin	0.00020499	1.452588	Up	+
6	2.115	$C_{10}H_{16}O_6$	233.1033	233.1025	3.4320	Glycerol 1-propanoate diacetate	0.00055778	1.428456	Down	+
7	2.246	$C_{12}H_{15}NO_4$	236.0928	236.0923	2.1178	N-Acetyl-L-phenylalanine	0.00065172	1.540518	Down	-
8	1.556	$C_{10}H_{18}N_2O_5$	245.1144	245.1137	2.8558	gamma-Glutamylvaline	0.014577	1.058267	Up	-
9	12.535	$C_{16}H_{30}O_2$	253.2174	253.2168	2.3695	Palmitoleic acid	0.025726	1.083636	Up	-
10	13.094	$C_{17}H_{32}O_2$	267.2322	267.2324	-0.7484	10Z-Heptadecenoic acid	0.011613	1.191439	Up	-
11	11.224	$C_{18}H_{28}O_2$	275.2019	275.2011	2.9070	Stearidonic acid	0.0061486	1.273002	Up	-
12	6.282	$C_{17}H_{28}O_3$	279.1966	279.1960	2.1490	12S-HHT	0.00014577	1.685472	Down	-
13	14.101	$C_{19}H_{36}O_2$	295.2644	295.2637	2.3708	10(Z)-Nonadecenoic acid	0.013346	1.116053	Up	-
14	9.583	$C_{20}H_{32}O_2$	303.2326	303.2324	0.6596	Arachidonic acid	0.00017535	1.587549	Down	-
15	1.162	$C_{19}H_{19}FN_2O$	311.1572	311.1560	3.8566	N-Desmethylcitalopram	0.00036324	1.453125	Down	+
16	5.041	$C_{18}H_{32}O_4$	311.2228	311.2222	1.9279	13-L-Hydroperoxylinoleic acid	7.34E-07	1.756142	Up	-
17	4.691	$C_{20}H_{28}O_3$	315.1964	315.1960	1.2691	4-Hydroxyretinoic acid	3.67E-05	1.663752	Down	-
18	4.330	$C_{20}H_{30}O_3$	317.2123	317.2117	1.8915	15-KETE	4.82E-05	1.646914	Down	-
19	8.524	$C_{22}H_{30}O_2$	327.2334	327.2324	3.0559	15beta-Hydroxydesogestrel	0.00089866	1.500369	Down	+
20	8.747	$C_{22}H_{30}O_3$	341.2112	341.2117	-1.4654	2,3-Dinor-6-keto-prostaglandin F1 a	0.00024974	1.571711	Down	-
21	8.735	C21H30O5	363.2174	363.2172	0.5506	Cortisol	7.20E-05	1.606373	Down	+
22	7.414	$C_{22}H_{37}NO_3$	364.2864	364.2852	3.2941	20-HETE ethanolamide	0.0055586	1.336779	Down	+

23	12.836	$C_{21}H_{34}O_5$	365.2336	365.2328	2.1904	5alpha-Tetrahydrocortisol	5.27E-05	1.569726	Up	-
24	12.823	$C_{17}H_{33}O_7P$	379.1895	379.1886	2.3735	1-(9Z-tetradecenoyl)-glycero-3-phosphate	0.0085462	1.257764	Up	-
25	4.492	$C_{17}H_{22}O_{11}$	403.1251	403.1240	2.7287	4-Hydroxy-5-(3',4'-dihydroxyphenyl)-valeric acid-O-glucuronide	0.0012653	1.338344	Down	+
26	6.333	$C_{21}H_{44}NO_6P$	436.2822	436.2828	-1.3753	LysoPE(16:1)	0.0057345	1.154259	Up	-
27	6.315	$C_{23}H_{46}NO_6P$	464.3132	464.3141	-1.9383	LysoPE(18:2)	0.00063783	1.493280	Down	+
28	10.865	$C_{26}H_{52}NO_6P$	506.3618	506.3611	1.3824	LysoPC(18:1)	0.0027496	1.390476	Up	+
29	11.454	$C_{26}H_{56}NO_6P$	510.3922	510.3924	-0.3919	LysoPC(O-18:0)	4.82E-05	1.650555	Up	+
30	6.283	$C_{26}H_{48}NO_7P$	518.3241	518.3247	-1.1576	LysoPC(18:3)	0.0022099	1.329841	Down	+
31	9.112	$C_{28}H_{50}NO_7P$	544.3409	544.3403	1.1023	LysoPC(20:4)	0.0065085	1.480929	Down	+
32	8.704	$C_{28}H_{54}NO_7P$	548.3725	548.3716	1.6412	LysoPC(20:2)	2.52E-08	1.877385	Down	+
33	10.867	$C_{28}H_{56}NO_7P$	550.3877	550.3873	0.7268	PC(20:1)	0.0005869	1.427991	Down	+
34	11.761	$C_{30}H_{58}NO_7P$	576.4031	576.4029	0.3470	LysoPC(22:2)	0.0015706	1.341560	Up	+
35	6.541	$C_{29}H_{49}O_{12}P$	619.2886	619.2883	0.4844	LysoPI(20:4)	9.27E-05	1.642532	Up	-
36	2.381	$C_{33}H_{65}O_8P$	619.4344	619.4339	0.8072	PA(30:0)	8.12E-06	1.686100	Down	-
37	6.521	$C_{30}H_{45}N_6O_{16}P$	775.2563	775.2552	1.4189	Tetrahydromethanopterin	2.28E-06	1.762729	Up	-
38	6.990	$C_{40}H_{80}O_{13}P_2$	829.4985	829.4996	-1.3261	PGP(16:0/18:0)	7.00E-05	1.624328	Up	-
39	6.704	$C_{44}H_{74}NO_{12}P$	838.4861	838.4870	-1.0734	PS(DIME(9,5)/DIME(9,3))	0.0096785	1.186467	Up	-
40	7.687	$C_{61}H_{90}O_6$	919.6826	919.6816	1.0873	TG(58:14)	1.38E-06	1.709596	Down	+
41	2.365	$C_{47}H_{88}O_{16}P_2$	969.5482	969.5469	1.3408	PIP(38:2)	6.33E-05	1.576837	Down	-

Table S8. The regulated differential metabolites observed in urine of PRE-treated AD mice

NO.	Retention time (min)	Formula	Measured m/z	Theoretical m/z	Delta (ppm)	Metabolite	p value	VIP	Trend	ESI Model
1	3.212	$C_6H_{10}O_2$	113.0602	113.0603	-0.8845	2-Methyl-4-pentenoic acid	0.000296	1.394610	Up	-
2	5.421	$C_7H_8O_2$	123.0443	123.0446	-2.4381	3-Hydroxybenzyl alcohol	0.000003	1.723488	Down	-
3	2.360	$C_5H_7NO_3$	128.0343	128.0348	-3.9052	Pyroglutamic acid	0.000657	1.320667	Down	-
4	1.161	$C_6H_{13}NO_2$	148.0976	148.0974	1.3505	L-Isoleucine	0.004108	1.160460	Up	+
5	0.803	$C_4H_8N_2O_3$	131.0453	131.0457	-3.0524	Ureidopropionic acid	0.004448	1.156348	Down	-
6	2.784	$C_7H_{12}O_3$	143.0705	143.0708	-2.0969	3-Methyl-2-oxovaleric acid	4.75E-05	1.533251	Up	-
7	4.695	$C_5H_{11}NO_2S$	148.0435	148.0432	2.0264	L-Methionine	0.001166	1.215971	Up	-
8	0.706	$C_7H_{15}NO_3$	162.1135	162.1130	3.0843	L-Carnitine	6.61E-05	1.483532	Down	+
9	2.163	$C_9H_{11}NO_2$	164.0714	164.0712	1.2190	L-Phenylalanine	0.000000	1.453428	Up	-
10	8.134	$C_9H_{10}O_3$	165.0558	165.0552	3.6351	Phenyllactic acid	0.006135	1.459150	Down	-
11	3.105	$C_8H_8O_4$	167.0345	167.0344	0.5987	p-Hydroxymandelic acid	0.000001	1.605309	Down	-
12	8.187	$C_6H_8O_6$	175.0240	175.0243	-1.7140	Ascorbic acid	0.000005	1.528530	Up	-
13	2.358	$C_6H_{11}NO_3S$	176.0384	176.0381	1.7042	N-Formyl-L-methionine	0.000564	1.340784	Down	-
14	1.003	$C_7H_{11}NO_5$	188.0560	188.0559	0.5318	N-Acetyl-L-glutamic acid	1.68E-05	1.558789	Down	-
15	4.972	$C_{10}H_9NO_3$	190.0506	190.0504	1.0524	5-Hydroxyindoleacetic acid	0.000001	1.572726	Up	-
16	14.521	$C_{13}H_{20}O$	193.1594	193.1592	1.0354	2-trans,4-cis,7-cis-Tridecatrienal	4.85E-05	1.506385	Down	+
17	6.534	$C_{10}H_{11}NO_3$	192.0664	192.0661	1.5620	Phenylacetylglycine	0.000017	1.491088	Up	-
18	10.011	$C_{10}H_{17}NO_3$	198.1136	198.1130	3.0286	Ecgonine methyl ester	0.000374	1.371629	Up	-
19	12.890	$C_{13}H_{12}O_2$	199.0756	199.0759	-1.5070	Safynol	0.000065	1.410714	Up	-
20	10.660	$C_{10}H_{16}O_4$	199.0965	199.0970	-2.5113	Decenedioic acid	0.000195	1.385154	Up	-
21	9.124	$C_{11}H_{11}NO_3$	204.0662	204.0660	0.9801	Indolelactic acid	0.000010	1.571946	Down	-
22	8.081	$C_4H_8NO_7P$	211.9964	211.9960	1.8868	L-Aspartyl-4-phosphate	0.000527	1.291335	Up	-

23	10.330	$C_{10}H_{14}N_2O_4$	225.0871	225.0875	-1.7771	Porphobilinogen	0.000328	1.406704	Up	-
24	4.807	$C_{10}H_{17}NO_5$	230.1031	230.1028	1.3038	Suberylglycine	0.000033	1.469287	Up	-
25	10.591	$C_{12}H_{21}NO_4$	244.1550	244.1549	0.4096	Tiglylcarnitine	0.000016	1.400792	Up	+
26	1.984	$C_8H_{10}O_7S$	249.0074	249.0069	2.0080	Hydroxymethoxyphenylcarboxylic acid-O-sulphate	0.000018	1.534826	Down	-
27	11.191	$C_{13}H_{23}NO_4$	258.1706	258.1705	0.3873	2-Hexenoylcarnitine	1.35E-05	1.551802	Down	+
28	8.066	$C_{10}H_{14}N_2O_6$	257.0776	257.0774	0.7780	3-Methyluridine	0.000370	1.392829	Up	+
29	3.884	$C_9H_{10}O_7S$	261.0067	261.0069	-0.7663	Homovanillic acid sulfate	0.000041	1.471571	Up	-
30	4.542	$C_{15}H_{12}N_2O_3$	269.0931	269.0926	1.8581	4-Hydroxyphenytoin	8.95E-07	1.634942	Down	+
31	13.260	$C_{14}H_{27}NO_4$	274.2023	274.2018	1.8235	Heptanoylcarnitine	0.000055	1.401430	Up	+
32	8.215	$C_{13}H_{16}O_7$	283.0825	283.0818	2.4728	Cresol glucuronide	0.000010	1.489423	Up	-
33	2.526	$C_{13}H_{23}NO_6$	290.1611	290.1604	2.4125	3-Methylglutarylcarnitine	2.90E-07	1.698193	Up	+
34	5.655	$C_{11}H_{19}NO_9$	308.0987	308.0982	1.6229	N-Acetylneuraminic acid	0.002963	1.369443	Up	-
35	9.630	$C_{20}H_{28}O_5$	347.1856	347.1859	-0.8641	12,20-Dioxo-leukotriene B4	0.000347	1.440886	Up	-
36	8.358	$C_{19}H_{30}O_5S$	371.1894	371.1892	0.5388	Androsterone sulfate	4.87E-05	1.508746	Up	+
37	14.482	$C_{21}H_{37}NO_5$	384.2761	384.2750	2.8625	3-Hydroxy-5, 8-tetradecadiencarnitine	9.45E-05	1.456379	Down	+
38	12.191	$C_{19}H_{32}O_8$	387.2023	387.2019	1.0331	9,13-Dihydroxy-4-megastigmen-3-one 9-glucoside	2.12E-05	1.529389	Down	-
39	10.344	$C_{16}H_{26}O_{11}$	393.1398	393.1397	0.2544	1-(3-Methyl-2-butenoyl)-6-apiosylglucose	0.000070	1.517309	Up	-
40	11.581	$C_{26}H_{36}O_8$	477.2487	477.2488	-0.2095	Retinoyl b-glucuronide	0.000236	1.378044	Up	+
41	12.491	$C_{24}H_{47}O_9P$	509.2884	509.2879	0.9818	1-(9Z-octadecenoyl)-sn-glycero-3-phospho-(1'-sn-glycerol)	0.000046	1.457596	Up	-
42	6.534	$C_2H_5NO_2$	74.0244	74.0242	2.7018	Glycine	0.000038	1.442129	Up	-
43	0.784	$C_3H_7NO_2$	88.0402	88.0399	3.4075	beta-Alanine	5.43E-07	1.792002	Down	-

Table S9. The regulated differential metabolites observed in faeces of PRE-treated AD mice

NO.	Retention time (min)	Formula	Measured m/z	Theoretical m/z	Delta (ppm)	Metabolite	p value	VIP	Trend	ESI Model
1	2.601	C_9H_{12}	121.1015	121.1017	-1.6515	Isopropylbenzene	0.00022273	1.534845	Up	+
2	0.631	$C_2H_7NO_3S$	124.0069	124.0068	0.8064	Taurine	0.00069418	1.415219	Down	-
3	0.554	$C_7H_{19}N_3$	146.1658	146.1657	0.6842	Spermidine	0.0052738	1.249205	Down	+
4	1.594	$C_6H_{14}N_2O_3$	161.0932	161.0926	3.7246	5-Hydroxylysine	3.65E-08	1.561697	Down	-
5	0.652	$C_6H_{10}O_6$	177.0402	177.0399	1.6945	L-Gulonolactone	2.54E-07	1.521393	Down	-
6	3.076	$C_9H_{11}NO_3$	180.0665	180.0661	2.2214	L-Tyrosine	0.00018836	1.570137	Up	-
7	2.171	$C_7H_{16}N_4O_2$	189.1359	189.1352	3.7011	Homo-L-arginine	0.00030671	1.942624	Down	+
8	2.721	$C_9H_{18}N_2O_4$	217.1184	217.1188	-1.8423	Leucyl-Serine	1.68E-05	1.445341	Up	-
9	1.414	$C_{11}H_{22}N_2O_3$	229.1554	229.1552	0.8728	Val-Leu/Valyl-leucine	0.00063101	1.540030	Down	-
10	1.193	$C_{10}H_{20}N_2O_4$	231.1348	231.1345	1.2979	Threoninyl-Isoleucine	0.00066182	1.656026	Down	-
11	2.574	$C_{11}H_{14}N_2O_4$	237.0874	237.0875	-0.4218	Glycyltyrosine	7.79E-10	1.650781	Down	-
12	11.163	$C_{14}H_{28}O_3$	243.1965	243.1960	2.0560	3-hydroxytetradecanoic acid	2.64E-05	1.432696	Up	-
13	3.794	$C_7H_{14}N_2O_6S$	253.0486	253.0494	-3.1614	5-L-Glutamyl-taurine	5.42E-05	1.539362	Down	-
14	11.774	$C_{16}H_{32}O_3$	271.2280	271.2273	2.5809	alpha-Hydroxypalmitic acid	1.71E-06	1.515009	Up	-
15	9.044	$C_{18}H_{32}O_2$	279.2326	279.2324	0.7162	Linoleic acid	2.49E-05	1.405478	Up	-
16	3.902	$C_{10}H_{12}N_4O_6$	285.0841	285.0835	2.1046	Xanthosine	2.55E-06	1.756837	Down	+
17	5.220	$C_{20}H_{24}O_2$	297.1856	297.1855	0.3365	17a-Ethynylestradiol	0.00021562	1.528222	Down	+
18	1.103	$C_{17}H_{16}O_5$	299.0925	299.0920	1.6717	L-Cystathionine	5.10E-08	1.599195	Down	-
19	9.404	$C_{18}H_{39}NO_2$	302.3054	302.3059	-1.6540	Sphinganine	0.00019379	1.549820	Up	+
20	12.843	$C_{20}H_{34}O_2$	305.2487	305.2481	1.9656	Eicosatrienoic acid	0.0012189	1.582232	Up	-
21	5.014	$C_{18}H_{31}NO_4$	324.2186	324.2175	3.3928	10-Nitrolinoleic acid	9.43E-06	1.713278	Down	-
22	8.113	$C_{20}H_{30}O_4$	335.2226	335.2222	1.1932	Prostaglandin B2	1.49E-05	1.679782	Down	+

23	3.884	$C_{12}H_{21}N_3O_8$	335.1339	335.1329	2.9839	Aspartylglycosamine	1.28E-05	1.727806	Up	+
24	10.105	$C_{21}H_{36}O_4$	353.2684	353.2692	-2.2646	MG(18:3)	2.67E-06	1.510449	Up	+
25	10.993	$C_{21}H_{37}NO_4$	366.2654	366.2644	2.7303	Leu-Leu-Leu	9.61E-07	1.491269	Down	-
26	10.992	$C_{18}H_{35}N_3O_4$	357.2633	357.2628	1.3995	3, 5-Tetradecadiencarnitine	7.75E-06	1.431014	Down	+
27	12.167	$C_{21}H_{41}NO_4$	372.3121	372.3114	1.8801	Tetradecanoylcarnitine	1.28E-07	1.576278	Down	+
28	11.354	$C_{24}H_{38}O_3$	373.2753	373.2743	2.6790	3b-Hydroxy-5-cholenoic acid	2.02E-05	1.435735	Up	-
29	9.204	$C_{24}H_{38}O_4$	389.2684	389.2692	-2.0551	Nutriacholic acid	7.59E-05	1.359716	Up	-
30	8.405	$C_{24}H_{40}O_4$	391.2846	391.2848	-0.5111	Deoxycholic acid	5.51E-06	1.473818	Down	-
31	10.841	$C_{25}H_{38}O_4$	401.2681	401.2692	-2.7413	7b-Hydroxy-3-oxo-5b-cholanoic acid	7.82E-07	1.537099	Up	-
32	4.641	$C_{24}H_{38}O_5$	405.2653	405.2641	2.9610	3,7-Dihydroxy-12-oxocholanoic acid	3.05E-05	1.418673	Down	-
33	6.021	$C_{24}H_{40}O_5$	407.2786	407.2798	-2.9464	Cholic acid	1.89E-06	1.524142	Down	-
34	5.176	$C_{21}H_{45}O_6P$	423.2869	423.2876	-1.6537	LPA(18:0)	7.05E-05	1.359288	Down	-
35	7.320	$C_{27}H_{46}O_5$	449.3261	449.3267	-1.3353	Coprocholic acid	2.12E-06	1.466962	Up	-
36	3.102	$C_{30}H_{48}O_9$	553.3384	553.3377	1.2651	Lithocholate 3-O-glucuronide	0.0013286	1.372070	Down	+
37	3.922	$C_{33}H_{42}N_4O_6$	591.3185	591.3183	0.3382	Urobilin	1.12E-05	1.723172	Down	+
38	3.821	$C_{33}H_{46}N_4O_6$	595.3499	595.3496	0.5039	Stercobilin	1.57E-10	1.993985	Up	+
39	6.551	$C_{33}H_{48}N_4O_6$	597.3650	597.3652	-0.3348	Stercobilinogen	7.46E-09	1.934742	Up	+
40	4.604	$C_{33}H_{56}O_{10}$	611.3984	611.3795	30.9137	Cholestane-3,7,12,25-tetrol-3-glucuronide	8.25E-09	1.936611	Up	-
41	5.486	$C_{36}H_{38}N_4O_8$	655.2759	655.2768	-1.3735	Coproporphyrin I	6.63E-05	1.613202	Up	+
42	5.563	$C_{45}H_{84}O_{16}P_2$	942.5163	942.5156	0.7427	PIP(36:2)	0.00013095	1.715069	Down	-

REFERENCES

1. Bromley-Brits, K., Deng, Y.,Song, W. Morris Water Maze Test for Learning and Memory Deficits in Alzheimer's Disease Model Mice. *J Vis Exp*, 2011;
2. Lueptow, L.M. Novel Object Recognition Test for the Investigation of Learning and Memory in Mice. *J Vis Exp*, 2017;
3. Zhang, M., Qian, C., Zheng, Z.G., et al. Jujuboside A promotes Abeta clearance and ameliorates cognitive deficiency in Alzheimer's disease through activating Axl/HSP90/PPARgamma pathway. *Theranostics*, 2018; 8: 4262-78.
4. Tayeb, H.O., Yang, H.D., Price, B.H., et al. Pharmacotherapies for Alzheimer's disease: Beyond cholinesterase inhibitors. *Pharmacology & Therapeutics*, 2012; 134: 8-25.
5. Baik, S.H., Kang, S., Lee, W., et al. A Breakdown in Metabolic Reprogramming Causes Microglia Dysfunction in Alzheimer's Disease. *Cell metabolism*, 2019; 30: 493-507.e6.
6. Heneka, M.T., Carson, M.J., Khoury, J.E., et al. Neuroinflammation in Alzheimer's disease. *The Lancet Neurology*, 2015; 14: 388-405.
7. Singh, P., Gollapalli, K., Mangiola, S., et al. Taurine deficiency as a driver of aging. *Science*, 2023; 380:
8. Nasaruddin, M.L., Hölscher, C., Kehoe, P., et al. Wide-ranging alterations in the brain fatty acid complement of subjects with late Alzheimer's disease as detected by GC-MS. *American Journal of Translational Research*, 2016; 8: 154-65.
9. Kao, Y.-C., Ho, P.-C., Tu, Y.-K., et al. Lipids and Alzheimer’s Disease. *Int J Mol Sci*, 2020; 21:
